# The impact of structured self-monitoring of blood glucose on clinical, behavioral, and psychosocial outcomes among adults with non-insulin-treated type 2 diabetes: a systematic review and meta-analysis

**DOI:** 10.3389/fcdhc.2023.1177030

**Published:** 2023-04-20

**Authors:** Elizabeth Holmes-Truscott, Shaira Baptista, Mathew Ling, Eileen Collins, EIif I. Ekinci, John Furler, Virginia Hagger, Jo-Anne Manski-Nankervis, Caroline Wells, Jane Speight

**Affiliations:** ^1^ School of Psychology, Deakin University, Geelong, VIC, Australia; ^2^ The Australian Centre for Behavioural Research in Diabetes, Diabetes Victoria, Melbourne, VIC, Australia; ^3^ Neami National, Preston, VIC, Australia; ^4^ Diabetes Victoria, Melbourne, VIC, Australia; ^5^ Department of Medicine, Austin Health, Melbourne Medical School, The University of Melbourne, Melbourne, VIC, Australia; ^6^ The Australian Centre for Accelerating Diabetes Innovations (ACADI), The University of Melbourne, Melbourne, VIC, Australia; ^7^ Department of General Practice, The University of Melbourne, Melbourne, VIC, Australia; ^8^ School of Nursing and Midwifery, Faculty of Health, Deakin University, Burwood, VIC, Australia; ^9^ Diabetes Tasmania, Hobart, TAS, Australia

**Keywords:** self-monitor of blood glucose (SMBG), structured self-monitoring of blood glucose, HbA1c, behavioral outcomes, psychological outcome, systematic review, meta-analysis, type 2 diabetes

## Abstract

**Background:**

Self-monitoring of blood glucose (SMBG) is considered of little clinical benefit for adults with non-insulin-treated type 2 diabetes, but no comprehensive review of a structured approach to SMBG has been published to date.

**Purpose:**

To conduct a systematic review and meta-analysis of the impact of sSMBG on HbA1c, treatment modifications, behavioral and psychosocial outcomes, and; examine the moderating effects of sSMBG protocol characteristics on HbA1c.

**Data sources:**

Four databases searched (November 2020; updated: February 2022).

**Study selection:**

Inclusion criteria: non-randomized and randomized controlled trials (RCTs) and prospective observational studies; reporting effect of sSMBG on stated outcomes; among adults (≥18 years) with non-insulin-treated type 2 diabetes. Studies excluded if involving children or people with insulin-treated or other forms of diabetes.

**Data extraction and analysis:**

Outcome data extracted, and risk of bias/quality assessed independently by two researchers. Meta-analysis was conducted for RCTs, and moderators explored (HbA1c only).

**Data synthesis:**

From 2,078 abstracts, k=23 studies were included (N=5,372). Risk of bias was evident and study quality was low. Outcomes assessed included: HbA1c (k=23), treatment modification (k=16), psychosocial/behavioral outcomes (k=12). Meta-analysis revealed a significant mean difference favoring sSMBG in HbA1c (-0·29%, 95% CI: -0·46 to -0·11, k=13) and diabetes self-efficacy (0.17%, 95% CI: 0.01 to 0.33, k=2). Meta-analysis revealed no significant moderating effects by protocol characteristics.

**Limitations:**

Findings limited by heterogeneity in study designs, intervention characteristics, and psychosocial assessments.

**Conclusion:**

A small positive effect of sSMBG on HbA1c and diabetes self-efficacy was observed. Narrative synthesis of sSMBG intervention characteristics may guide future implementation.

**PROSPERO registration:**

https://www.crd.york.ac.uk/prospero/display_record.php?ID=CRD42020208857, identifier CRD42020208857.

## Introduction

Persistent hyperglycemia among adults with type 2 diabetes is a risk factor for diabetes-related complications (1), and indicative of the need to optimize glucose management (2). However, there is evidence demonstrating sub-optimal diabetes management, including diet and physical activity (3, 4), inconsistent and non-persistent use of prescribed diabetes medications (5); and routine delay of clinically-indicated treatment intensification, including insulin (6, 7). For adults with non-insulin-treated type 2 diabetes, clinical guidelines focus on glycated hemoglobin (HbA1c) as the preferred form of glucose monitoring to inform clinical recommendations (1, 2). Routine self-monitoring of blood glucose (SMBG) is considered “of limited additional clinical benefit while adding to burden and cost” (8). While HbA1c is an important clinical indicator of the risk of long-term complications, it does not capture everyday glucose variability, which is an independent risk factor for complications (9). Furthermore, HbA1c has limited utility for informing or evaluating specific behavioral changes, is not well understood by people with type 2 diabetes (10–12), does not highlight episodes of hypoglycemia (which can occur in those taking sulphonylureas (2)), and may be inaccurate in the context of kidney disease, anemia, pregnancy and hemoglobinopathies (13).

While SMBG and continuous glucose monitoring (CGM) enable glucose monitoring in real-time, they are complex interventions, requiring skills to monitor, interpret, and take appropriate action of glucose levels, underpinned by diabetes education and health professional support (14). SMBG protocols need to be individualized, considering, for example, frequency, intensity, and duration of monitoring (15). However, published reviews have concluded that SMBG is clinically ineffective (16–18), and people with type 2 diabetes report SMBG as painful, time consuming, frustrating, demoralizing, and unhelpful (12, 19). Furthermore, SMBG is not cost-effective in people with non-insulin-treated type 2 diabetes (20). These findings have informed clinical practice guidelines and policy, which now recommend that SMBG not be used routinely in this group (21–26). CGM may overcome some of the perceived challenges of SMBG (e.g. pain and inconvenience of monitoring *via* finger prick device; resources required to identify glucose trends), but there remains limited evidence for the impact or acceptability of CGM among people with non-insulin-treated type 2 diabetes (27) and recommendations for its use among people with type 2 diabetes are limited to those administering insulin (28).

Previous reviews of the evidence for SMBG in adults with non-insulin-treated type 2 diabetes have been critiqued for the heterogeneity of studies included in terms of population as well as intervention characteristics (12, 21). Typically, trials have investigated the effect of routine *unstructured* SMBG (uSMBG), i.e. *ad-hoc* monitoring, which provides insufficient data to inform appropriate decisions and actions by either the person with type 2 diabetes or the health professional (12). In the past decade, trials have examined the utility of a *structured* approach to SMBG (sSMBG) (12, 21). sSMBG is an episodic, intensive, short-term approach (e.g. seven datapoints daily for a three-day period), which involves identifying and reflecting on glucose patterns, making behavioral changes (e.g. to diet, activity, medication taking) to improve glucose levels, and repeating the cycle to evaluate the effectiveness of the changes (21). Targeted use of sSMBG may be consistent with clinical guidelines, which recommend SMBG use in particular circumstances, e.g. when HbA1c is above target or treatment modification is required (15, 25),. Such targeted use of sSMBG may be both clinically- and cost-effective (21, 29, 30).

Recent meta-analyses (29, 30) have examined the clinical efficacy of sSMBG compared to uSMBG, among adults with non-insulin-treated type 2 diabetes. They identified greater improvement in HbA1c associated with sSMBG (29, 30), particularly when monitoring data informed medication adjustments (29). However, inclusion criteria specified randomized controlled trials (RCTs) including a uSMBG comparator (k=3 (29) and k=4 (30)), thus overlooking evidence where sSMBG was compared to usual care, no SMBG, or CGM. Furthermore, previous reviews have paid little attention to the impact of sSMBG on treatment modification, behavioral outcomes (i.e. self-care) or psychosocial outcomes (e.g. self-efficacy, treatment satisfaction, well-being, quality of life) (17, 29, 31). Finally, despite wide variation in the operationalization of sSMBG, there has been no investigation of the moderating effect of intervention characteristics (e.g. frequency, intensity and duration of monitoring) on the impact of sSMBG.

Our aim was to conduct a systematic review and meta-analysis to determine the effectiveness of sSMBG, among adults with non-insulin-treated type 2 diabetes, on clinical, behavioral, and psychosocial outcomes. Our secondary aim was to examine the extent to which operationalization of sSMBG (i.e. intervention characteristics) moderates the effect on HbA1c.

## Methods

This study includes a comprehensive systematic review of heterogenous trials and prospective observational studies, as well as a meta-analysis of RCTs comparing sSMBG to unstructured or usual care. Our reporting is guided by the PRISMA (Preferred Reporting Items for Systematic Reviews and Meta-Analyses) checklist (32), and the review protocol was pre-registered (PROSPERO: CRD42020208857; https://www.crd.york.ac.uk/prospero/display_record.php?ID=CRD42020208857).

### Search strategy and selection criteria

Studies were deemed eligible for the systematic review if they examined the impact of sSMBG among adults (≥18 years) with non-insulin-treated type 2 diabetes on ≥1 of the outcomes of interest; and were RCTs, non-randomized trials or single-arm prospective observational studies. Outcomes assessed were clinical (i.e. HbA1c, diabetes medication prescription/changes); behavioral (e.g. physical activity, diet, medication taking), and; psychosocial (e.g. emotional well-being, diabetes self-efficacy). As the operationalization of sSMBG varies, we defined this as “SMBG conducted in a purposeful, pre-specified pattern over a pre-specified timeframe, involving at least paired monitoring” (i.e. ≥2 timepoints). Studies were excluded if they involved children, people with other forms of diabetes (type 1, gestational), or only those with insulin-treated diabetes.

We conducted a systematic search of four databases (EMBASE, Medline Complete (EBSCOhost), CINAHL Complete (EBSCOhost), APA PsycInfo (EBSCOhost) on 20^th^ November 2020, and updated it on 7^th^ February 2022. The search strategy was developed by SB and EHT, in consultation with JS. Search terms combined keywords and relevant controlled vocabulary terms relating to type 2 diabetes (population) and sSMBG (intervention). The following limits were applied in all databases: Language (English) and year of publication (1^st^ January 2000 to 6^th^ February 2022). The reference lists of relevant published reviews were also searched for eligible studies (17, 29, 31).

Duplicates were identified and removed *via* a combination of ‘deduplicator’ software (https://www.xrmtoolbox.com/plugins/DynCrmExp.Deduplicator/), deduplication functions in EndNote and COVIDENCE, as well as *via* manual review. Titles and abstracts of were screened independently by EHT and SB, and relevant full-text articles retrieved and assessed independently for eligibility by EHT and SB. At each stage, any disagreement was resolved through mutual discussion.

### Data extraction and analysis

Data were extracted from the included articles using a pre-set, study-specific template, including:

1. study characteristics: e.g., design, setting, country/s, eligibility criteria, trial duration, follow-up, sample size, attrition, participant characteristics.
2. intervention descriptions and fulfilment: e.g., sSMBG protocol, metric tracking; supportive training for interpretation, healthcare professional feedback and/or responsive action, pre-specified and observed intervention fulfilment.
3. outcomes: raw group statistics for clinical, behavioral and psychosocial outcomes. Where self-report tools used, only validated scales considered.

Two researchers (EHT, SB and/or ML) extracted outcome data independently. Discrepancies were discussed and resolved. Study authors were contacted regarding missing data. Data that were provided in figures only, were determined using a WebPlotDigitiser program by ML or SB (33–37).

A narrative synthesis was conducted for all eligible studies, describing study characteristics, intervention details, and the main findings of interest. For RCTs comparing sSMBG to a “usual care” comparator (i.e. no SMBG or uSMBG), a random-effects meta-analysis (using a restricted maximum likelihood estimation method in the metafor package for R (38), version 3.0-3.2) was conducted to examine the absolute mean difference in HbA1c (%). Standard errors and confidence intervals of means were converted to standard deviation scores (39), while median and inter-quartile range results were omitted. Two RCTs eligible for inclusion in the meta-analysis included multiple sSMBG intervention arms compared to one control condition (40, 41). Where sSMBG was implemented with co-interventions, all participants allocated to sSMBG were treated as a single arm (41). Where outcomes were reported separately for comparable sSMBG interventions (40), these results were treated as nested and modelled with a random intercept per study to account for non-independence of the estimates of the two treatment conditions. A Cochran’s Q test was conducted to examine whether variations in the observed effects were likely to be attributable solely to sampling error. Heterogeneity was assessed using the I² statistic for HbA1c. Meta-analyses were performed only where heterogeneity was <85%. Simple and multiple meta-regression were both performed to confirm the characteristics contributing to heterogeneity. Statistical significance was regarded as p<0.10. In addition, tests were conducted for moderation of the effect on HbA1c by analysis type (intention to treat (ITT) vs per protocol (PP)), comparator condition, and sSBMG intervention characteristics. Finally, meta-analyses were repeated for the following outcomes, for which meaningful data were extracted from at least two eligible RCTs (drawing on intention to treat or per protocol data, as available): intensified diabetes medication (n, %); insulin initiation (n, %); general emotional well-being; depressive symptoms; diabetes-specific distress; self-efficacy; and treatment satisfaction.

Risk of bias was assessed independently (by SB and a second researcher) for HbA1c using the Cochrane risk of bias tool for RCTs (39), and the quality of non-randomized trials and cohort studies was assessed using the National Heart Lung and Blood Institute quality assessment tools (42). Articles from the updated search were assessed by SB only.

## Results

**Figure 1:**
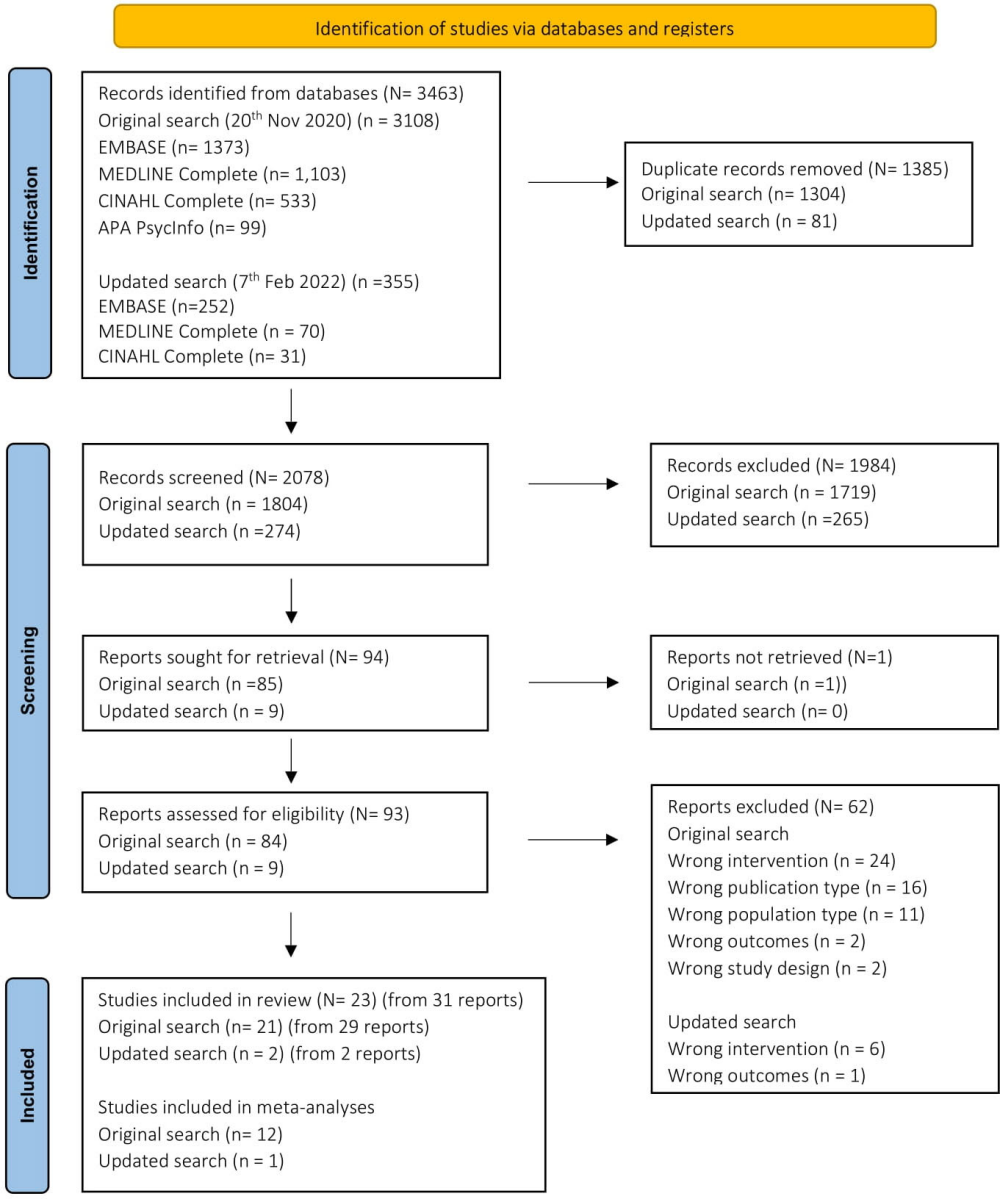
PRISMA Flowchart (32).

**Figure 1** shows the screening process, and reasons for exclusions. A total of n=31 articles were included, presenting the results of k=23 studies (some studies had multiple publications). Study and participant characteristics are summarized in **Supplementary Table S1**. Studies were conducted in 30 countries and included an overall sample of N=5,372 (range N=34 to N=1,024). Eighteen studies pre-specified an HbA1c eligibility criterion, with a lower limit range of 6% to 8% (42 – 64mmol/mol) (k=17) and an upper limit range of 8% to 13% (64 – 119 mmol/mol) (k=13).

Of the 20 eligible RCTs, most (k=16) compared one or more sSMBG interventions to a “usual care” control with or without uSMBG; two RCTs compared sSMBG to an “active SMBG control”, which for the purposes of this review are deemed “less intensive sSMBG” (i.e. two intervention arms), two compared sSMBG to CGM, one compared sSMBG to self-monitoring of urine glucose, and one compared three forms of sSMBG without a non-sSMBG control. Other studies included two prospective, observational single-arm studies (33, 43) and one retrospective (i.e. non-randomized) two-arm observational study (44). Study duration varied from three months (k=4) to 12 months (k=7), with three studies (2 RCTs, 1 prospective observational study) reporting relevant longer-term follow-up data from 18 to 36 months following trial conclusion (34, 45, 46). **Table 1** shows wide variation in the operationalization of sSMBG, along with pre-specified criteria for and observed intervention fulfillment.

**Table 1:** Summary of sSMBG intervention descriptions and intervention fulfilment.

Study Name (if specified) Author (year)	N checks per day	N profiles per active week	N active weeks/N study weeks	Tracking: BGL+ diet/physical activity/goals etc	Training: interpret/action		HCP facilitated feedback/action	Intervention fulfilment	
					PwT2D	HCP		Pre-specified criteria	Observations
Randomized Controlled Trials									
Bergenstal (2022) (47)	4 + 7x3 (once a month)	7	16/16	Yes	Yes	–	Yes	NR	NR
Bonomo (2010) (48)	Tx1:4 Tx2: 6	Tx1, Tx2: 1	Tx1: 6/26 Tx2: 13/26	–	–	–	Yes	≥70% of the required checks + recorded results	Tx1: 73% Tx2: 44% P<.001
Cox (2020) (49)	~8 (estimated: before/2 hours after nutrient intake + before/0.5 hours after sustained physical activity (>10minutes))	7	16/16	Yes	Yes	Yes	Yes	NR	NR
Davidson (2005) (50)	2	6	26/26	Yes	–	–	Yes	NR	NR
DiGEM Farmer (2007), French (2008), Simon (2008) (20, 40, 51)	Tx1, Tx2: 3	Tx1, Tx2:2	Tx1, Tx2:52/52	Tx1, Tx2: Yes	Tx1. Tx2: Yes	Tx1, Tx2: Yes	Tx1, Tx2: Yes	33% of required checks in first 3mths (≥26 checks)	Tx1: n=99, 67%
DINAMIC 1 Barnett (2008) (52)	5 (7/mth)	2 (+1/mth)	27/27	–	–	–	–	NR	NR
Greenwood (2015) (53)	2	7	12/12	–	Yes	Yes	Yes	100% (84 paired glucose checks over 12wks)	0% (range: 0-73 pairs)
IN CONTROL Malanda (2016) (54)	6	2	8/52	–	Yes	–	–	≥80% of required checks	Tx1: range: n=30-33, 50%-55.0%
Kan (2017) (55)	2	7	26/26	–	–	–	Yes	NR	NR
Li (2016) (35)	Tx1: 2 Tx2: 2 T3: 7	Tx1: 6 Tx2: 3 T3: 3	Tx1: 36/36 Tx2: 36/36 T3: ~8/36	–	Yes	–	Yes	75% of the required checks + blood test and clinic visits	Tx1: n=31, 72% Tx2: n=31, 79% Tx3: n=34, 85%
Ngaosuwan (2015) (56)	2	4	24/24	Yes	–	–	Yes	≥95% of the required checks	100%
Nishimura (2017) (57)	7	3	3/24	Yes	Yes	–	–	NR	NR
PRISMA Bosi (2013), Russo (2016) (58, 59)	Tx1, Tx2: 4	Tx1, Tx2: 3	Tx1: 2/52 Tx2: 52/52	–	Tx1: - Tx2: Yes	–	Tx1: - Tx2: Yes	Tx1: <200 unstructured checks over 12mths Tx2: ≥80% of the required checks	Tx1: n=98, 81% Tx2: n=200, 60%
ROSSO International Kempf (2013) (45)	7+	1	4/12	–	Yes	–	–	NR	NR
SMBG^a^ Parsons (2019) (41)	Tx1, Tx2: 4 (7/qtrly)	Tx1, Tx2: 2 (3/qtrly)	52/52	Yes	Yes	Yes	Tx1: Yes Tx2: Yes + telecare	≥80% of the required checks ≥80% paired-monitoring per day	Tx1: 69% Tx2: 74% Tx1: 77% Tx2: 83%
SMBG^b^, Schwedes (2002), Siebolds (2006) (36, 37)	6	2	26/26	Yes	Yes	Yes	Yes	≥70% of the required checks	NR
St Carlos Duran (2010), García de la Torre (2013) (46, 60)	Tx1, Tx2:6	NA*	NA*	Tx1: - Tx2: yes	–	–	Yes	NR	At 12mth: Tx1+Tx2: n=96, 97% performed sSMBG At 3yrs: Tx1: n=63, 97% Tx2: n=55, 85%
STeP Fisher (2011), Polonsky (2011)^a^, Polonsky (2011)^b^, Fisher (2012) sher (2012) (61, 62, 64, 65)	7	3	5/52	Yes	Yes	Yes	Yes	≥80% of the required checks + completed tracking tool and discussion of results at ≥4 of the 5 clinic visits.	n=130, 69%
ZODIAC Kleefstra (2010) (63)	4	2	52/52	–	–	–	–	≥80% of the required checks	n=7, 77%
ROSES Franciosi (2011) (66)	2	3	13/26	Yes	Yes	Yes	Yes	≥80% of the required checks	Tx1: 92.9%
Retrospective two-armed observational study									
Madeo (2020) (44)	3	2	26/26	–	–	–	–	>80% of the required checks	Tx1: n=22, 81%
Prospective observational single group study									
Cander (2015) (43)	2	4	13/13	–	–	–	–	NR	NR
ROSSO Kempf (2010), Kempf (2012) (33, 34)	7+	1	4/12	Yes	Yes	–	–	NR	NR

NA, Not applicable; Tx1, Treatment 1; Tx2,Treatment 2 *number of checks per active week and regularity of active weeks varied over study duration.

All 23 studies assessed the impact of sSMBG on HbA1c, 16 reported treatment modification outcomes (typically operationalized as n/% participants with diabetes medication type and/or dose change), and 12 reported behavioral or psychosocial outcomes. Psychosocial outcomes included general emotional well-being (k=5), depressive symptoms (k=5), diabetes treatment satisfaction (k=4), diabetes-specific distress (n=4), diabetes self-efficacy (k=5), generic health status (k=3) and diabetes-specific quality of life (k=2) with other assessments (e.g. glucose monitoring satisfaction, beliefs about illness) assessed in single studies only. Behavioral outcomes included self-reported diabetes self-management (k=4) (including medication taking and/or diet and physical activity).

Overall, some concern about the quality and or/risk of bias was observed for most studies assessing HbA1c (k=17/22) and behavioral/psychosocial outcomes (k=7/12), mainly pertaining to the randomization process, deviations from the intended intervention protocol, and selection bias in the reporting results (**Supplementary Figures S1** and **S2**).

### HbA1c

Among the 16 RCTs and one retrospective observational study comparing sSMBG intervention(s) with usual care (with/without uSMBG), eight studies identified a between-group difference in HbA1c at follow-up favoring the intervention (ITT=3; PP=5) (**Supplementary Table S2**). Two RCTs examined long-term follow-up data, both reporting a sustained effect (ROSSO International: 3m RCT, 18m follow-up (45); St Carlos: 12m RCT, 36m follow-up) (46). In the two prospective observational studies, small significant reductions were observed in HbA1c from baseline to study end (33, 34, 43).

**Figure 2:**
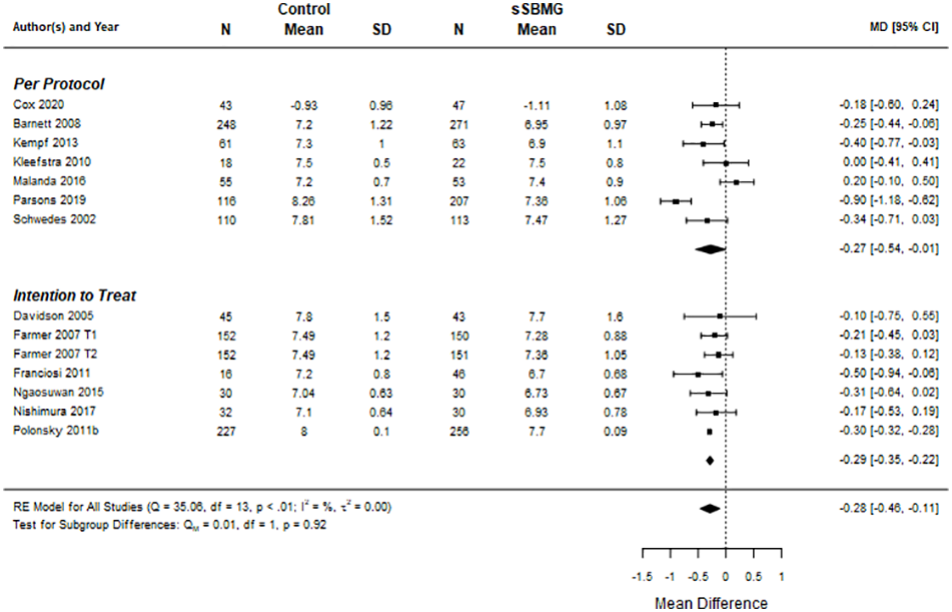
Forest plot of meta-analysis for effect of sSMBG versus control (usual unstructured or no SMBG) on HbA1c (%). Data are mean difference (95% confidence internal). Note. DiGEM (51) includes two treatment arms relative to control referred to as Farmer 2007 T1 and T2. NA, Not applicable.

**Figure 2** shows the meta-analysis for HbA1c (k=13 eligible RCTs, i.e. with usable data). Overall, the between-group mean difference in HbA1c across trials was –0.28% (95% CI: -0.46 to -0.11), favoring sSMBG. Both ITT and PP meta-analyses provided statistically meaningful mean differences in HbA1c between interventions. The Cochrane Q test (=35, p=0.0008) and I^2^ (82.1%) suggest statistically significant heterogeneity.

Meta-analysis findings were not moderated by comparator (unstructured SMBG vs no SMBG). Elsewhere, no significant between-group differences in HbA1c were reported at follow-up in the two RCTs comparing sSMBG with CGM (47, 49), nor in the single RCT comparing sSMBG with urine monitoring (54). Two of three comparative effectiveness trials (comparing two or more sSMBG interventions in the absence of a control arm) reported a modest but statistically significant between-group difference in HbA1c at follow-up (35, 48, 58). The PRISMA study identified that greater clinical benefit was derived with more intensive sSMBG (i.e. four checks per day, three days per week, in addition to participant training and HCP support to interpret sSMBG profiles) than with less intensive sSMBG (i.e. four checks per day, twice a year with no additional training or support) (58). Li et al. (35) identified greater improvement in HbA1c among those performing monthly seven-point sSMBG profiles on three consecutive days relative to monitoring weekly, twice per day, on three consecutive days, but comparable to those performing paired monitoring six days per week (35). Meta-analysis revealed no statistically significant effects on HbA1c of sSMBG intervention protocol in terms of checks per day, number of active weeks, profiles per week, additional tracking beyond blood glucose levels, interpretation training or health professional feedback/action.

### Healthcare professionals’ prescriptions of diabetes medications

Five of k=13 RCTs reported a significant between-group difference in diabetes treatment modifications at follow-up (**Supplementary Table S3**). This included four studies that identified a greater percentage of participants receiving medication recommendations/prescriptions in the sSMBG arm relative to “usual care”, and one study that observed greater medication change among participants undertaking “intensive sSMBG” relative to less intensive sSMBG (originally termed “active control”) (58). One study (of a potential k=4) reported a significant between-group difference in insulin uptake during the study, favoring sSMBG (61). However, meta-analysis (k=5, 6 comparisons) revealed no significant effect on initiation of insulin (-0.13, 95% CI: -1.61 to 1.35), or oral diabetes medication changes (0.44, 95% CI: -0.12 to 0.10).

### Psychosocial and behavioral outcomes

Of the 12 studies reporting psychosocial and/or behavioral effects of sSMBG, few demonstrated significant benefits or detrimental impacts (**Supplementary Table S4**). Based on meta-analysis of eligible RCTs, sSMBG was associated with significantly improved diabetes-specific self-efficacy (k=2, 0.17 95% CI: 0.01 to 0.33), but there was no significant effect for general emotional well-being, depressive symptoms, diabetes distress or diabetes treatment satisfaction (**Table 2**).

**Table 2:** Summary of main effect of sSMBG (compared to non-sSMBG) on psychosocial outcomes for eligible RCTs.

Outcomes	Studies (k)	Pooled sample (n)	RE model			Overall effect	
			Q (df)	I^2^	P value	SMD (95% CI)	P value
Generic							
General emotional well-being	4^*^ (37, 51, 62, 63)	1,198	5.06 (4)	NA	0.28	-0.04 (-0.21 to 0.13)	0.67
Depressive symptoms	3 (37, 54, 62, 63)	814	78.42(2)	97.05%	<0.001	-0.59 (-1.51 to 0.32)	0.20
Diabetes-specific							
Diabetes distress	2 (54, 62)	591	152.05 (1)	99.34%	<0.001	-0.91 (-3.66 to 1.85)	0.52
Diabetes self-efficacy	2 (54, 62)	591	0.14 (1)	0.00%	0.70	0.17 (0.01 to 0.33)	0.03
Diabetes treatment satisfaction	4* (37, 51, 54, 63)	823	3.68 (4)	NA	0.45	0.00 (-0.14 to 0.14)	0.98

*k=4 studies, but outcomes draw on five comparisons, as DiGEM (51) includes two treatment arms relative to control. NA, Not applicable.

The DiGEM study identified a small, significant between-group difference in health status (EQ-5D) (20). Worse outcomes were observed among participants allocated to the more intensive sSMBG intervention arm (but not the less intensive arm) relative to “usual care”, likely owing to significant increases in anxiety and depressive symptom scores. In contrast, per protocol analysis of the STeP study showed a significant between-group difference, favoring sSMBG, for general emotional well-being (WHO-5), regimen-related diabetes distress (DDS subscale), and diabetes-specific self-efficacy (CIDS) (61, 62, 64). Subgroup analyses revealed greatest reduction in depressive symptoms and diabetes distress among those in the intervention arm who reported elevated depressive symptoms at baseline (62). Examining three sSMBG protocols, Li et al. (35) reported significantly greater improvement in general emotional well-being (WHO-5) and reduction in diabetes distress (PAID) among participants performing monthly seven-point profiles, or paired monitoring three days per week, relative to those performing more frequent paired monitoring (six days per week). In one study, intervention group participants showed significantly greater improvement in general (but not diabetes-specific) quality of life than those in the control group (p<0.001) (55).

Regarding self-reported diabetes self-management behaviors (**Supplementary Table S4**), two of four studies measuring relevant outcomes reported between-group comparisons. Both RCTs identified significant differences in ‘healthful eating’ at follow-up favoring the control arm, while no change was observed in physical activity or medication-taking in either study (51, 57). Elsewhere, Cox et al. determined no between-arm differences in objective physical activity data (obtained *via* pedometer), nor carbohydrate or caloric intake when comparing participants undertaking a behavioral intervention in combination with sSMBG, CGM or usual SMBG (49).

## Discussion

This systematic review identified 23 studies investigating the impact of sSMBG on HbA1c, treatment modifications and/or psychosocial and behavioral outcomes among adults with non-insulin-treated type 2 diabetes. Meta-analysis of 13 RCTs identified a statistically significant, although clinically small, mean difference in HbA1c, favoring sSMBG relative to usual care. This finding is consistent with previous reviews (29, 30). Meta-analysis also revealed a modest, but significant, mean difference in diabetes self-efficacy (i.e. confidence in self-managing diabetes) associated with sSMBG, potentially explaining how sSMBG can lead to improvement in HbA1c (67–69). No other differential psychosocial/behavioral outcomes were identified across studies, nor a proportionate difference in prescription of diabetes medications overall, or for insulin specifically. However, narrative review identified sSMBG was associated with treatment modification in five RCTs, negative impact (on general health status) in one study, and both general and diabetes-specific psychosocial benefits in one study, where data were explored per protocol (i.e. those who engaged with the intervention). Overall, there was considerable heterogeneity in study comparators and designs, as well as the operationalization of sSMBG, though there were no moderating effects of intervention characteristics, analysis type, or study comparator (uSMBG vs. no SMBG) on HbA1c. Finally, there are a paucity of studies investigating CGM relative to sSBMG among adults with non-insulin-treated type 2 diabetes, with limited evidence that CGM has benefit for HbA1c over manual sSMBG.

The lack of a well-defined and consistent operationalization of sSMBG limits the ability to specify exactly what ‘effective sSMBG’ is and is not. Despite the lack of moderation effects observed, narrative comparison of intervention features of the eight ‘effective’ and the seven ‘ineffective’ sSMBG interventions (relative to non-sSMBG) suggests trends in protocol variation, which require further investigation. Effective sSMBG interventions, as indicated by a statistically significant effect on HbA1c, typically incorporated the following characteristics:

- self-monitoring of glucose at least four times per day, and commonly, the application of semi-regular, seven-point profiles
- an active role for the person with diabetes in tracking additional data (e.g. diet, physical activity) and/or provision of training to interpret sSMBG data
- training of the healthcare professional in the interpretation of sSMBG profiles; *and*
- action by the healthcare professional in terms of interpretation, feedback, and/or responsive treatment modification recommendations.

The latter is consistent with the findings of two prior meta-analyses, which identified greater clinical benefit when SMBG was followed by responsive treatment modifications (29, 30). However, this was not observed in the current study likely due to the broader inclusion criteria. Further research is warranted to better ascertain the effect of *supportive* sSMBG (i.e. including tracking, training, and feedback/action), as well as its feasibility for real-world clinical practice, given the considerable variation, where reported, in intervention protocol fulfilment. Indeed, per protocol analysis of some studies suggests greater reduction in HbA1c and improvement in psychosocial/behavioral outcomes among those able to sustain the sSMBG protocol. Furthermore, previous research suggests that adults with non-insulin-treated type 2 diabetes lack understanding of how to interpret and respond to SMBG data, and healthcare professionals do not use SMBG data to guide clinical discussion and treatment recommendations (70). A narrative review of research exploring the experience of sSMBG implementation among adults with non-insulin-treated type 2 diabetes, and their healthcare professionals, may be warranted to answer clinically relevant research questions, such as for whom sSMBG may be most feasible, acceptable and beneficial, and the extent to which equity of access to sSMBG plays a role in outcomes. Similarly, such unanswered questions remain for the use, acceptability, accessibility, and effect of CGM in this population.

This review is the first to investigate the impact of sSMBG on psychosocial/behavioral outcomes, and thus addresses an important gap in the literature. While psychosocial data were incorporated in their 2012 Cochrane review (17) of the effectiveness of SMBG overall, limited conclusions could be drawn at that time and greater attention to such outcomes in future RCTs was called for. Of the thirteen sSMBG RCTs identified subsequently (**Supplementary Table S5**), eight reported assessment of psychological/behavioral constructs. Although our review identified conflicting evidence, typically there was minimal impact of sSMBG on general and diabetes-specific psychosocial or behavioral outcomes, relative to control. Further, cautious interpretation is necessary where an overall effect was observed: meta-analysis of diabetes-specific self-efficacy drew on the data of only two eligible RCTs, including one reporting per protocol results only. Variation in psychosocial and behavioral measures used (15 constructs measured with 24 different validated tools) may explain, in part, the lack of an effect. Consensus on the operationalization of core outcomes, and their further assessment using validated measures, remains necessary (67–69). Consideration needs to be given to using measures identified in previous studies to enable further cross-study comparisons, as well as to answering novel research questions. Importantly, a key gap in the sSMBG evidence is investigation of participants’ experiences of and/or satisfaction with glucose monitoring, which was assessed only by Cox et al. (49) which is likely to have explanatory value in terms of persistence with sSMBG in real-world type 2 diabetes self-management.

This systematic review has several strengths. It is the most comprehensive review to date of sSMBG among adults with non-insulin-treated type 2 diabetes, presenting robust consideration of the available evidence for the effectiveness of sSMBG on HbA1c, treatment modifications, psychosocial and behavioral outcomes, updating and extending the evidence described in previous reviews (17, 29, 31). In comparison to three major meta-analyses of SMBG RCTs published in the past decade (17, 29, 31), the current review identified six studies not previously incorporated (**Supplementary Table S5**). Further, while those reviews were limited to RCTs only, this review incorporated evidence from multiple quantitative study designs, including RCTs, non-RCTs, and prospective and retrospective observational studies. The available evidence was examined using both narrative synthesis and, where possible, meta-analysis (RCTs only). Our approach supplemented the evidence for the effectiveness of sSMBG with detailed extraction and analysis of study design, intervention elements, and protocol fulfillment, which may influence the engagement of both the person with diabetes and their healthcare professionals, and the effectiveness of sSMBG. Therefore, the recommendations made are also informed by valuable contextual information, which offers potential explanations for the observed data trends.

This review also has limitations. First, the evidence base has several shortcomings. Most notably, few studies reported outcomes beyond HbA1c. Among those that did, there was little consistency in how those outcomes were operationalized. This meant limited opportunity for comparison between, and synthesis across, studies. In addition, the reliability of the findings may be limited by the heterogeneity of eligible sSMBG interventions (including disparate use of HbA1c and/or sSMBG to inform clinical management), variation in control conditions (i.e. CGM, non-SMBG, sSMBG, uSMBG)), the risk of bias/quality of these, and the inclusion of non-RCT study designs. Second, we excluded from our meta-analyses one RCT, which we determined to be a comparative effectiveness trial (i.e. comparing two sSMBG arms), despite the original authors describing an “active control” (58, 59). This exclusion may have impacted the overall effect size. However, this decision was considered appropriate given the similarity of that ‘control’ arm to some sSMBG ‘intervention’ arms in other trials. Third, there remain unanswered questions regarding the optimization of sSMBG for this cohort. For example, while the current analyses showed no differential effects on HbA1c by active intervention duration (i.e. number of active weeks over trial duration), meta-analysis could not examine overall time effects nor could we examine predictors of sustained effect. Further, the current study did not assess the differential impact of sSMBG by baseline Hba1c. For several trials the pre-specified HbA1c inclusion criteria included a lower limit of ≤7.5%, and mean Hba1c at baseline varied widely (6.6% – 8.9%; **Supplementary Table S2**). Future research might explore the beneficial effects (and costs) of sSMBG for those with the highest clinical priority (i.e. above target HbA1c), for whom health gains are also most likely in terms complication risk reduction. Finally, the current review did not consider studies published in a language other than English, examine cost-effectiveness, consider clinical outcomes beyond HbA1c (e.g. hypoglycemia, glucose variability), or qualitative research which may answer clinically relevant research questions.

In conclusion, this comprehensive systematic review and meta-analysis demonstrates that structured SMBG (relative to usual care: unstructured or no SMBG) has a modest, but significant benefit for HbA1c and diabetes self-efficacy. There is limited evidence for a positive effect on treatment modification, behavioral or other psychosocial outcomes. However, the identified studies are highly heterogeneous. While meta-analyses did not identify any moderating effect of intervention characteristics on HbA1c, narrative synthesis suggests that the features of effective sSMBG intervention include: intensive (4-point) profiles with proactive data tracking, pro-active interpretation, feedback and modification of diabetes management, including medications and diet/activity. Evidence-based operationalization of sSMBG is warranted in both clinical practice and future research.

## Guarantor statement

EH-T is the guarantor of this work and, as such, had full access to all the data in the study and takes responsibility for the integrity of the data and the accuracy of the data analysis.

## Prior presentation

Data have been reported in brief (<300 word abstract) at an academic conference:

*Australasian Diabetes Congress*, Brisbane, Australia (8-10 August, 2022): Oral presentation

## Data availability statement

The original contributions presented in the study are included in the article/**Supplementary Material**. Further inquiries can be directed to the corresponding authors.

## Author contributions

EH-T and JS developed the review questions and protocol with input from all co-authors. SB and EH-T developed the search with input from the broader team. SB ran the searches. EH-T and SB screened the abstracts and full-text articles to identify eligible studies. SB, EH-T, and ML extracted the data. SB conducted the risk of bias and quality assessments. ML ran the meta-analysis with input from EH-T and SB. EH-T and SB prepared the manuscript with input from all co-authors. All authors contributed to the article and approved the submitted version.
