# Supplementary material for: The impact of structured self-monitoring of blood glucose on clinical, behavioral, and psychosocial outcomes among adults with non-insulin-treated type 2 diabetes: a systematic review and meta-analysis
Source: Front Clin Diabetes Healthc. 2023 Apr 20;4:1177030. doi: 10.3389/fcdhc.2023.1177030 (PMC10157033; doi:10.3389/fcdhc.2023.1177030)
Supplement: Supplementary file 1 [file DataSheet_1.docx]

# Supplementary Tables

## Supplementary Table S1: Study and participant characteristics

| **Study name  (if specified)  Author (year) Country** | **Study arms and description^** | **Trial duration and follow-up** | **Sample size:  Total (N) and by sub-sample (n) and/or trial arm (n)** | **Inclusion criteria: HbA1c, mmol/mol (%)** | **Participant characteristics:* Age, years; Gender, %, Diabetes duration, years**† | **Attrition by study arm (n)** | **Relevant study outcomes: Primary and Other** |
| --- | --- | --- | --- | --- | --- | --- | --- |
| **Randomised Controlled Trials** | | | | | | | |
| Bergenstal (2022)(47) US | Tx1: sSMBG Tx2: CGM | 16 weeks | Total sample N=114 (Tx1: n=55, Tx2: n=59) Non-insulin-treated sub-sample (N=63):  Incretins: N=29 (Tx1: n=15, Tx2: n=14) Sulfonylurea: N=34 (Tx1: n=17, Tx2: 17) | ≥53 (≥7.0) | Total sample Age  Tx1: 58.8±10.0, Tx2: 59.3±8.9 Gender: Female  Tx1: 58%, Tx2: 49% Diabetes duration Tx1: 12.7±7.0, Tx2: 11.8±6.5 Non-insulin-treated sub-sample (-) | Total sample Tx1: n=3 Tx2: n=4 | Primary: HbA1c Other: Treatment modification (Diabetes medication change) |
| Bonomo (2010)(48) Italy | Tx1: less intensive sSMBG Tx2: more intensive sSMBG | 6 months | N=273 (Tx1: n= 96, Tx2: n= 177) | n/a | Age  Tx1: 62.8±9.8, Tx2: 65.3±9.6  Gender: Female  Tx1: 37(-), Tx2: 69(-)  Diabetes duration  Tx1: 10.4±8.5, Tx2: 10.8±7.9 | Tx1: n=0  Tx2: n=0 | Primary: HbA1c Other: Treatment modification (Diabetes medication / dose change) |
| Cox (2020)(49) US | C: usual care (usual SMBG) Tx1: sSMBG Tx2: CGM | 3 months | Total sample N=172; sample considered: N=132 (C: n=36, Tx1: n=50, Tx2: n=46) | ≥51 (≥6.8) | Age Tx1: 54.8±11.6, Tx2: 58.2±11.9, C: 54.7±11.7 Gender: Female Tx1: 63.8%, Tx2: 51.2%, C: 69.7% Diabetes duration Tx1: 4.8±3.2, Tx2: 5.7±3.2, C: 6.1±3.1 | C: n=3 Tx1: n=3  Tx2: n=3 | Primary: HbA1c Other: Depressive symptoms (PHQ-9) Diabetes distress (DDS, emotional and regimen subscales) Diabetes-specific self-efficacy (DSES) Glucose monitoring satisfaction (GMSS) Treatment modification (Medication Effect Score; MES) |
| Davidson (2005)(50) US | C: Usual care (no SMBG) Tx1: sSMBG | 6 months | N=88 (C: n=45, Tx1: n=43) | n/a | Age  C: 49.8±11.2, Tx1: 50.9±11 Gender: Female  C: 31 (67%), Tx1: 34 (79%) Diabetes duration  C: 5.5±4.7), Tx1: 5.8±5.8 | C: n=1, Tx1: n=0 | Primary: HbA1c Other: Treatment modification (Medications prescribed) |
| DiGEM  Farmer (2007), French (2008), Simon (2008)(20,40,51) UK | C: usual care (no SMBG)  Tx1: less intensive sSMBG  Tx2: more intensive sSMBG | 12 months | N=453 (C: n=152, Tx1: n=150, Tx2: n=151) | ≥44  (≥6.2) | Age= 65.7 (-)  Gender: Male  C: 85 (55.9%), Tx1: 88 (58.7%), Tx2: 87 (57.6%))  Diabetes duration  3(2-7) | C: n=18, Tx1: n=14, Tx2: n=25 | Primary: HbA1c  Other: Beliefs about medication (BMQ)  Beliefs about self-monitoring (study-specific)  Diabetes-specific self-management behaviour (SDSCA, MARS)  Diabetes treatment satisfaction (DTSQ)  General health status (EQ-5D)  General psychological well-being (W-BQ12)  Illness perceptions (IPQ-R)  Treatment modification (Diabetes medication/dose change; insulin starts) |
| DINAMIC 1  Barnett (2008)(52)  Czech Republic, Hungary, Iran, Malaysia, Poland, Slovakia and  Turkey | C: usual care (no SMBG)  Tx1: sSMBG | 27 weeks | N=610 (C: n=299  Tx1: n=311) | 53 -86  (7-10) | Age  C = 56.1±9.1, Tx1: 55.9±9.3  Gender: Male  C: 155 (51.8%), Tx1: 150 (48.2%)  Diabetes duration  C: 2.8±3.7, Tx1: 2.8±4.5 | C: n=47, Tx1: n=37 | Primary: HbA1c |
| Greenwood (2015)(53)  US | C: Usual care (usual SMBG)  Tx1: sSMBG + telecare | 6 months | N=90 (C: n=45, Tx1: n=45) | 58 – 96 (7.5-10.9) | Age  C: 57.5±10.6, Tx1:53.9±10.4  Gender: Female  C: 19 (25%), Tx1: 23 (25%)  Diabetes duration (-) | C: n=3, Tx1: n=4 | Primary: HbA1c  Other: Diabetes knowledge (DKT)  Diabetes-specific self-efficacy (DES-SF)  Diabetes self-management behaviours (SDSCA)  Treatment modification (Diabetes medication change) |
| IN CONTROL  Malanda (2016)(54)  Netherlands | C: usual care (unspecified SMBG)  Tx1: sSMBG  Tx2: Self-monitoring of urine glucose (SMUG) | 12 months | N=181 (C: n=62, Tx1: n=60, Tx2: n=59) | ≥53  (≥7) | Age  C: 61.2±8.1, Tx1: 60.8±7.5, Tx2: 62.7±7.7  Gender: Male  C: 42 (68%), Tx1: 43 (72%), Tx2: 35 (59%)  Diabetes duration  C: 7 (4-9), Tx1: 6 (3-8), Tx2: 7 (3-9) | C: n=7, Tx1: n=7, Tx2: n=16 | Co-primary: Diabetes distress (PAID)  Diabetes-specific self-efficacy (CIDS-2)  Other: Depressive symptoms (PHQ-9)  HbA1c  Treatment modification (Diabetes medication/dose change; insulin starts)  Diabetes treatment satisfaction (DTSQ) |
| Kan (2017)(55)  China | C: Usual care (usual SMBG)  Tx1: sSMBG | 6 months | Total sample  N=250 (C: n=129, Tx1: n=121)  Non- insulin treated sub-sample  N=120 (C: n=60, Tx1: n=60) | ≥ 64  (≥8) | Total sample  Age  C: 58.7±10.8, Tx1:56.6±11.1  Gender: Male  C: 78 (60.5%), Tx1: 74 (61.6%)  Diabetes duration  C: 12.8±7.0, Tx1: 10.6±7.2  Non-insulin treated sub-sample (-) | Total sample  C: n=8, Tx1: n=10  Non-insulin treated sub-sample (-) | Primary: HbA1c  Other: Diabetes-specific quality of life (ADDQoL-Chinese version) |
| Li (2016)(35)  Taiwan | Tx1: six-pair sSMBG  Tx2: three-pair sSMBG  Tx3: seven-point sSMBG | 36 weeks | N=122 (Tx1: n=43, Tx2:n=39, Tx3: n=40) | 53 - 108 (7-12) | Age  Tx1: 58.9±9.8, Tx2: 58.4±8.3, Tx3: 58.7±10.1  Gender: Male Tx1: 23 (-), Tx2: 15 (-), Tx3: 20 (-)  Diabetes duration  Tx1: 8.4±5.9, Tx2: 9.1±6.3, Tx3: 10.1±7.0 | Tx1: n=12,  Tx2: n=9, Tx3: n=9 | Primary: HbA1c  Other: Depressive symptoms (CES-D)  Diabetes distress (PAID)  Diabetes-specific self-efficacy (PDSMS)  General emotional wellbeing (WHO-5)  Treatment modification (Diabetes medication change) |
| Ngaosuwan (2015)(56)  Thailand | C: usual care (unspecified SMBG)  Tx1: sSMBG + individualised nutritional therapy | 24 weeks | N=60 (C: n=30, Tx1: n=30) | 48 – 80 (6.5-9.5) | Age  C: 59.1±7.9, Tx1: 52.1±10.2  Gender: Male  C: 10 (33%), Tx1: 10(33%)  Diabetes duration  C: 4.5 (1.8-8.0), Tx1: 2 (1-5.8) | C: n=0, Tx1: n=0 | Primary: HbA1c |
| Nishimura (2017)(57) Japan | C: Usual care (usual SMBG)  Tx1: sSMBG | 24 weeks | N=62 | 42 – 86 (6-10) | Age  C: 65.8±8.5, Tx1: 66.7±12.5  Gender: Female  C: 40.6%, Tx1: 36.7%  Diabetes duration  C: 14.1±8.7, Tx1: 13.3±8.4 | C: n=0, Tx1: n=0 | Primary: HbA1c  Other: Diabetes self-management behaviour (SDSCA) Treatment modification (OHA medication/dose change) |
| PRISMA  Bosi (2013), Russo (2016)(58,59)  Italy | Tx1: less intensive sSMBG  Tx2: intensive sSMBG | 12 months | N=1024 (Tx1: n= 523, Tx2: n=501) | 53 -75  (7-9) | Age  Tx1: 60.4 (54-68), Tx2: 60.2 (55-67)  Gender: Female  Tx1: 209 (40%), Tx2: 198 (39.5%)  Diabetes duration  Tx1: 6.2 (3.4-8.8), Tx2: 6.2 (3.2-8.8) | Tx1: n=71,  Tx2: n=70 | Primary: HbA1c  Other: Diabetes-specific locus of control (DsLOC)  Diabetes-specific quality of life (DSQoL)  Treatment modification (Diabetes medication change) |
| ROSSO International  Kempf (2013)(45)  Bulgaria | C: Usual care (no SMBG)  Tx1: sSMBG | 3 months, 18 months | N=124 (C: n=61,  Tx1: n=63) | n/a | Age  C: 58.9±9.8, Tx1: 56±8.4  Gender (nr)  Diabetes duration  C: 3.8±1.3, Tx1: 3.0±0.5 | 3 months  C: n=0, Tx1: n=0  1.5 years  C: n=1, Tx1: n=1 | Primary: HbA1c  Other: Treatment modification (Metformin dose) |
| SMBG^a^  Parsons (2019)(41)  UK | C: Usual care (no SMBG)  Tx1: sSMBG  Tx2: sSMBG + TeleCare | 12 months | N=446 (C: n=151,  Tx1: n=147, Tx2: n=148) | 58 – 119 (7.5-13) | Age  C: 60.7±11.0, Tx1: 62.9±9.3, Tx2: 61.6±9.8  Gender: Male  C: 88 (58%), Tx1: 82 (56%), Tx2: 88 (60%)  Diabetes duration  1 to 3 years: C: 30 (20%), Tx1: 20 (14%), Tx2: 28 (19%); 3 to 5 years: C: 29 (19%), Tx1: 32 (22%), Tx2: 23 (16%); 5 to 10 years: C: 53 (35%), Tx1: 47 (32%), Tx2: 55 (37%); >10 years: C: 39 (26%), Tx1: 48 (33%), Tx2: 42 (28%) | C: n=70,  Tx1: n=96,  Tx2: n=80 | Primary: HbA1c  Other: Treatment modification (Diabetes medication increase) |
| SMBG^b^  Schwedes (2002),  Siebolds (2006)(36,37)  Germany, Austria | C: Usual care (no SMBG)  Tx1: sSMBG | 6 months | N=250 (C: n=110,  Tx1: n=113) | 58 – 86 (7.5-10) | Age  C: 60.5±6.6), Tx1: 58.7±7.6  Gender: Male  C: 51.8%, Tx1: 52.2%  Diabetes duration (-) | NR | Primary: HbA1c  Other: Diabetes treatment satisfaction (DTSQ)  General well-being (PWQ) |
| St Carlos  Duran (2010), GarcíaDeLaTorre (2013)(46,60)  Spain | C: Usual care (usual SMBG)  Tx1: sSMBG  Tx2: sSMBG + supervised exercise | 12 months, 36 months | N=195 (C: n=65, Tx1: n=65, Tx2: n=65) Tx1 and Tx2 combined: n=99 (PP analysis) | <64  (<8) | Age  C: 57.5±10.1, Tx1: 58.6±13.6, Tx2: 58.2±13.2  Gender: Male  C: 27 (-), Tx1: 35 (-), Tx2: 32 (-)  Diabetes duration (-) | C: n=4, Tx1: n=0, Tx2: n=6 | Primary: HbA1c  Other: Treatment modification (Metformin prescription) |
| STeP  Fisher (2011), Polonsky (2011)^a^, Polonsky (2011)^b^,  Fisher (2012)(61-64)  US | C: Usual care (usual SMBG)  Tx1: sSMBG | 12 months | N=483 (C: n=227, Tx1: n=256) | 58 – 108 (7.5-12) | Age  C: 57±11.2), Tx1: 54.8±10.1  Gender: Male  C: 122 (53.7%), Tx1: 135 (52.7%)  Diabetes duration  C: 7.7±6.1, Tx1: 7.5±6.1 | C: n=40, Tx1: n=68 | Primary: HbA1c  Other: Depressive symptoms (PHQ-8)  Diabetes-related autonomous motivation (DRAM)  Diabetes self-management (IPAQ)  Diabetes distress (DDS)  Diabetes-specific self-efficacy (CIDS-2)  General emotional well-being (WHO-5)  Treatment modification (Diabetes medication and/or lifestyle modification recommendation; insulin starts) |
| ZODIAC  Kleefstra (2010)(65)  Netherlands | C: Usual care (no SMBG)  Tx1: sSMBG | 12 months | N=41 (C: n=19, Tx1: n=22) | 53 – 69 (7-8.5) | Age  C: 58.7±7.8, Tx1: 59.5±8.0  Gender: Male  C: 13 (72%), Tx1: 12 (55%)  Diabetes duration  C: 8 (3.8-11.3), Tx1: 5 (4-7) | C: n=1,  Tx1: n=0 | Primary: HbA1c  Other: Diabetes symptoms (DSC-r)  Diabetes treatment satisfaction (DTSQ)  General emotional well-being (WHO-5)  General health status (SF-36) |
| ROSES  Franciosi (2011)(66)  Italy | C: Usual care (unspecified SMBG)  Tx1: sSMBG | 6 months | N=62 (C: n=16, Tx1: n= 46) | 53 – 75 (7-9) | Age  C: 48.7±0.6, Tx1: 48.9±0.5  Gender: Male  C: 14 (87.5%), Tx1: 32 (69.6)  Diabetes duration  C: 3.2±4.4, Tx1: 3.4±3.5 | C: n=1, Tx1: n=4 | Primary: HbA1c  Other: Treatment modification (Diabetes medication /dose change) |
| **Retrospective two-armed observational study** | | | | | | | |
| Madeo (2020)(44)  Italy | C: Usual care (unspecified SMBG)  Tx1: sSMBG | 6 months | N=54 (C: n=20, Tx1: n=19) | n/a | Age  C: 62.7±9.6, Tx1: 65±7.7  Gender: Male  C: 13 (65%), Tx1: 12 (63%)  Diabetes duration  C: 9.8±9.1, Tx1: 10.6±6.8 | C: n=7, Tx1: n=8 | Primary: HbA1c |
| **Prospective observational single group study** | | | | | | | |
| Cander (2015)(43)  Turkey | Tx1: sSMBG | 3 months | N=34 (OHA group) | 58 – 86 (7.5-10) | Age = 53±9.7  Gender: Male = 9 (26.5%)  Diabetes duration= 6.6±5.8 | n=4 | Primary: HbA1c |
| ROSSO  Kempf (2010), Kempf (2012)(33,34)  Germany | Tx1: sSMBG | 12 weeks, 24 months | N=405 | n/a | Age = 58.2±9.3  Gender: Male = 161 (40%)  Diabetes duration= 2±1.5 | 3 months: n=78  24 months: n=99 | Primary: HbA1c  Other: Depressive symptoms (CES-D)  General health status (SF-36)  Treatment modification (OHA prescription) |

*Data listed is for total sample where not provided by treatment arm in original publication; ^^^arms reported where relevant to the current study, †data are Mean±SD, Median(IQR), or n(%)
C= Control, IQR= Interquartile Range, M= Mean, Med= Median, SD= Standard Deviation, Tx1= Treatment 1, Tx2= Treatment 2, Tx3= Treatment 3, NR= not reported, NA= Not applicable , OHA= Oral Hypoglycaemia Agent, PP= per protocol.
BMQ: Beliefs about Medicines Questionnaire, CES-D: Center for Epidemiologic Studies Depression scale, CIDS-2: Confidence in Diabetes Self-Care questionnaire, CN-ADDQoL: Chinese Normal Audit of Diabetes-Dependent Quality of Life, DDS: Diabetes Distress Scale, DES / DES-SF: Diabetes Empowerment Scale / DES short form, DKT: Diabetes Knowledge Test, DRAM: Diabetes-Related Autonomous Motivation assessed by 6 item subscale of Treatment Self-Regulation Scale, DSC-r: Diabetes Symptoms Checklist, DsLOC: Diabetes-specific Locus of Control, DSQoL: Diabetes-specific Quality of Life, DTSQ: Diabetes Treatment Satisfaction Questionnaire, EQ-5D: EuroQol EQ-5D, GMSS: Glucose Monitoring Satisfaction Scale, IPAQ: International Physical Activity Questionnaire, IPQ-R: Revised Illness Perception Questionnaire, MARS: Medication Adherence Report Scale, MES: Medication Effect Score, PAID: Problem Areas in Diabetes scale, PDSMS: Perceived Diabetes Self-Management Scale, PHQ-8/9: Patient Health Questionnaire- 8/9 item, PWQ: Patient Well-being Questionnaire, SDSCA: Summary of Diabetes Self-Care Activities, SF-36: Short-Form 36 Health Survey Questionnaire, WBQ-12: Well-Being Questionnaire, WHO-5: WHO five-item Well-being Index.

## Supplementary Table S2: HbA1c at baseline and follow-up, change from baseline and between-group difference at follow-up

| **Study name (if specified)**  **Author (year)** | **Study design** | **HbA1c (%) at baseline** | **HbA1c (%) at follow-up** | **Change from baseline** | **Between-group difference at**  **follow-up** |
| --- | --- | --- | --- | --- | --- |
| Bergenstal (2022) (47)^+^ | RCT  16w | Incretin group  Tx1: 7.6±0.2  Tx2: 7.8±0.2  Sulfonylurea group  Tx1: 7.8±0.3  Tx2: 8.3±0.3 | Incretin group  Tx1: 7.0±0.1  Tx2: 6.5±0.2  Sulfonylurea group  Tx1: 7.0±0.1  Tx2: 7.0±0.1 | NR | NR |
| Bonomo (2010) (48) | RCT  6m | Tx1: 8.04±0.80  Tx2: 8.06±0.82 | TX1: 7.79±1.02  Tx2: 7.83±0.91 | TX1: -0.25, p=0.06  Tx2: -0.23, p=0.0029 | NS  p=0.74 |
| Cox (2020) (49) | RCT  3m | C: 8.4 ±1.2Tx1: 8.1±1.1  Tx2: 8.6±1.6 |  | C: -0.85±1.54  Tx1: -1.11±1.08  Tx2: -0.93±0.96 | NS  p=0.13 |
| Davidson (2005) (50) | RCT  6m | C: 8.4±2.1  Tx1: 8.5±2.2 | C: 7.8±1.5  Tx1: 7.7±1.6 | C: -0.6±2.1  Tx1: -0.8±1.6 | NS  p=0.58 |
| DiGEM  Farmer (2007), French (2008),  Simon (2008) (20,40,51) | RCT  12m | C: 7.49±1.09  Tx1: 7.41±1.02  Tx2: 7.53±1.12 | C: 7.49±1.20  Tx1: 7.28±0.88  Tx2: 7.36±1.05 | C:-0.00±1.02  Tx1: -0.14±0.82  Tx2: -0.17±0.73 | NS  p=0.12 |
| DINAMIC 1  Barnett (2008) (52)* | RCT  27w | C: 8.12±0.84  Tx1: 8.12±0.89 | C: 7.2±1.22  Tx1: 6.95±0.97 | C: -0.91±1.29  Tx1: -1.15±1.14 | Significant: -0.25 (1.09), 95% CI, 0.06-1.03, p=0.0097 |
| Greenwood (2015) (53)* | RCT  6m | C: 8.16±1.1  Tx1: 8.46±1.1 | C: 7.46  Tx1: 7.35 | NR | NS  –0.11, p=0.55 |
| IN CONTROL  Malanda (2016) (54)*^ | RCT  12m | C: 7.4±0.6  Tx1: 7.5±0.5  Tx2: 7.7±1.0 | C: 7.2±0.7  Tx1: 7.4±0.9  Tx2: 7.3±0.8 | C: -0.2±0.6  Tx1: -0.1±0.9  Tx2: -0.4±1.2 | NS  Tx1 vs C: -0.0 (-0.2 to 0.1)  Tx2xC: 0.1 (-0.2 to 0.3)  Tx1 vs Tx2: -0.2 (-0.5 to 0.1) |
| Kan (2017) (55) | RCT  6m | NR for non-insulin sample | NR for non-insulin sample | C: -1.35±1.82, p<0.001  Tx1: -1.91±1.9, p<0.001 | NS  p=0.123 |
| Li (2016) (35) | RCT  36w | Tx1: 8.68±1.28  Tx2: 8.58±1.25  Tx3: 8.92±1.52 | Tx1: 7.08±0.86  Tx2: 7.36±0.84  Tx3: 7.1±0.63 | Tx1: p<0.05  Tx2: p<0.05  Tx3: p<0.05 | Significant only for T3xTx2: p<0.05 |
| Ngaosuwan (2015) (56) | RCT  24w | C: 7.14±0.52  Tx1: 7.56±0.74 | C: 7.04±0.63  Tx1: 6.73±0.67 | C: -0.20 (-0.13 to 0.45), p=0.283^#^  Tx1: -0.85 (0.18 to 1.33), p<0.001 | Significant  p<0.001 |
| Nishimura (2017) (57) | RCT  24w | C: 7.21±0.51  Tx1: 7.21±0.75 | C: 7.10±0.64  Tx1: 6.93±0.78 | C: -0.11 NS  Tx1: -0.28, p<0.05, 95% CI, -5.8 to -0.5 | NS |
| PRISMA  Bosi (2013), Russo (2016) (58,59) | RCT  12m | Tx1: 7.3±6.9-7.8  Tx2: 7.4±6.9-7.8 | Tx1: NR  Tx2: NR | Tx1: -0.27  Tx2: -0.39 | Significant: -0.12% (95% CI, -0.210 to -0.024; p= 0.013) |
| ROSSO international  Kempf (2013) (45)* | RCT  3m  2y | C: 7.5±1.0  Tx1: 7.4±1.6 | 3 months: C: 7.3±1.0; Tx1:6.9±1.1  1.5 years: C: 7.5±0.7; Tx1: 6.9±0.9 | 3 months: C: -0.2±0.6; Tx1: -0.5±0.9  1.5 years: C: +0.1 (nr); Tx1: -0.5±1.4 | Significant  3 months: p<0.001  1.5 years: p<0.05* |
| SMBGa  Parsons (2019) (41)* | RCT  12m | C: 8.65±1.07  Tx1: 8.53±1.08  Tx2: 8.63±1.15  Tx1+Tx2: 8.58±1.12 | C: 8.26±1.31  Tx1: 7.39±1.22  Tx2: 7.33±0.89  Tx1+Tx2: 7.36±1.06 | C: -0.30±-0.52 to -0.07  Tx1: -1.05±-1.29 to -0.8  Tx2: -1.17±-1.40 to -0.94 | Significant  Tx1 vs C: -0.75 (1.08 to -0.42), p<0.0001  Tx2 vs C: -0.87 (-1.19 to -0.55), p<0.0001  NS  Tx2 vs Tx1: -0.12 (-0.45 to 0.21), p=0.477 |
| SMBGb  Schwedes (2002),  Siebolds (2006) (36,37)* | RCT  6m | C: 8.35±0.75  Tx1: 8.47±0.86 | C: 7.81±1.52  Tx1: 7.47±1.27 | C: -0.54±1.41  Tx1: -1.0±1.08 | Significant  p=0.0086 (95% CI; 0.11–0.77) |
| St Carlos  Duran (2010),  GarcíaDeLaTorre (2013) (46,60) * | RCT  12m  3y | C: 6.6 (6.4-7.1)^#^  Tx1 + Tx2: 6.6 (5.8-7)^#^  Tx1: 6.6±0.3  Tx2: 6.7±0.5 | C: 6.6 (6.2-7.3)  12 months: Tx1 + Tx2: 6.1 (5.8-6.5)  3 years: Tx1: 6.2±0.6; Tx2: 6.1±0.6 | NR | Significant  12 months: Tx1+Tx2xC: p<0.01  3 years: Tx1 vs C: p<0.001; Tx2xC: p<0.001 |
| STeP  Fisher (2011), Polonsky (2011)^a^, Polonsky (2011)^b^, Fisher (2012) (61-64) | RCT  12m | C: 8.9±1.2  Tx1: 8.9±1.2 | C: 8.0±0.1  Tx1: 7.7±0.09 | C: -0.9±0.10^  Tx1: -1.2±0.09 | Significant  Tx1 vs C: -0.3, p=0.04 |
| ZODIAC  Kleefstra (2010) (65)* | RCT  12m | C: 7.7±0.4  Tx1: 7.6±0.5 | C: 7.5±0.5  Tx1: 7.5±0.8 | C: -0.1±0.9  Tx1: -0.7±0.8 | NS: Tx1 vs C: -0.05 (95% CI, ‑0.51 to 0.41, p=0.51) |
| ROSES  Franciosi (2011) (66) | Pilot RCT  6m | C: 7.9±0.6  Tx1: 7.9±0.2 | C: 7.2±0.2  Tx1: 6.7±0.1 | C: -0.7 (95% CI, -0.9 to ‑0.4)  Tx1: -1.2 (95% CI, -1 to ‑1.3) | Significant: -0.5 (95% CI, -0.9 to -0.0; p=0.04) |
| **Retrospective two-armed observational study** | | | | | |
| Madeo (2020) (44) | RO  6m | C: 8.5±1.0  Tx1: 8.4±0.6 | C: 7.7±1.0  Tx1: 7.5±0.7 | C: -0.8±1.1, p<0.01  Tx1: -0.9±0.7, p<0.001 | NS  p=0.620 |
| **Prospective observational single group study** | | | | | |
| Cander (2015) (43) | PO  3m | Tx1: 8.0±0.8 | Tx1: 7.6±1.1 | p= 0.011 | NA |
| ROSSO  Kempf (2010), Kempf (2012) (33,34)* | PO  3m  2y | Tx1 (Total sample, N=405): 6.7±1.0  Tx1 (3m completers, N=327): 6.7±1.0  Tx1 (2y completers, N=228): 6.7±1.0 | Tx1 (3m, Total sample) 6.5±0.8  Tx1 (3m completers only) 6.4±0.7  Tx1 (2y completers only) 6.6±0.8 | Tx1 (3m, Total sample) ‑0.2  Tx1 (3m completers) -0.3  Tx1 (2y completers) -0.1  Significant at 3m only p<0.001 | NA |

Data are mean±SD, unless otherwise indicated. *per protocol results, # Median (IQR), ^mean±SE, ^+^ Data determined using web plot digitizer, RCT= Randomised controlled trial, PO= Prospective observational study, RO= Retrospective 2-arm observational study, m=month, y=year, C= Control, Tx1= Treatment 1, Tx2= Treatment 2, Tx3= Treatment 3, NA = Not applicable, NR=not reported, NS=not significant

## Supplementary Table S3: Treatment modification outcomes (change at follow-up)

| **Study name (if specified)**  **Author (year)** | **Treatment modification outcome(s)** | **Results, by outcome** | **Significance** |
| --- | --- | --- | --- |
| **Randomised Controlled Trials** | | | |
| Bergenstal (2022) [47] | Medication change | NR | NR |
| Bonomo (2010) [48]* | Medication/dose change overall: % | Tx1: 47%, Tx2: 54% | Between groups: NS |
| Cox (2020) [49] | Medication/dose change overall: MES Scores at 3-months follow up | C: 1.31±0.76  Tx1: 1.13±0.80  Tx2: 1.13±0.87 | Between groups: NS |
| Davidson (2005) [50] | Medications prescribed: % | Metformin alone, C: 22%, Tx1: 19%; Metformin + sulfonylurea, C:38%, Tx1:35%; Triple oral therapy, C:27%, Tx1:37%; Insulin + pills, C: 13%,  Tx1: 7%; Insulin alone, C: 0; Tx1: 2% | Between groups: NS |
| DiGEM  Farmer (2007), French (2008), Simon (2008) [20,40,51] | Medication/dose increase overall: % | C: 30%, Tx1: 29%, Tx2: 32% | Between groups: NS |
|  | Initiated insulin: n | C: 1, Tx1: 4, Tx2: 5 | NR |
| Greenwood (2015)[53]* | Medication change: n | C: n=1, Tx1: n=27 | NR |
|  | Initiated insulin: n | C: n=0, Tx1: n=4 | NR |
| IN CONTROL, Malanda (2016)[54]* | Medication/dose change overall: % | NR | Between groups: NS |
|  | Initiated insulin: n | C: n=4, Tx1: n=3, Tx2: n=6 | Between groups:NS |
| Li (2016) [35] | Medication change: % | Tx1:37%, Tx2:23%, Tx3:32.5% | Between groups: NS |
| Nishimura (2017) [57] | OHA medication/dose increase: n, % | C: n=7, 22%, Tx1: n=15, 50% | Between groups: p<.05 |
| PRISMA, Bosi (2013), Russo (2016) [58,59] | Medication change: % | V2, 12w: Tx1:28%, Tx2:39%  V3, 24w: Tx1:20%, Tx2:32%  V4, 36w: Tx1:20%, Tx2:32%  V5, 52w: Tx1:17%, Tx2:22% | Between groups: p<.001  p<.001  p<.001  NS |
| ROSSO International, Kempf (2013) [45] | Metformin dose mg/day: mean increase | 12m, nr  18m follow up, C: 318 ± 474 mg/day, Tx1: 448 ±710 mg/day | Between groups: NS |
| SMBGa, Parsons (2019) [41]* | Total diabetes medication number increase: n, % | C: n=32, 28%, Tx1: n=45, 45.5%, Tx2: n=54, 50% | Between groups: p<.001 for Tx1 and Tx2 compared with C |
| St Carlos, Duran (2010), GarcíaDeLaTorre (2013) [46,60]* | Metformin prescription (alone): n, % | C: n=37, 60%, Tx1: n=64, 65% | Between groups: Modifications made earlier p<.002 and more frequently p<.001 |
|  | Glinides: n, % | C: n=9, 14%, Tx1: n=4, 4% |  |
|  | Sulphonylureas: n, %, | C: n=13, 21%, Tx1: n=1, 1% |  |
|  | Pioglitazone: n, % | C: NR, Tx1: n=7, 7% |  |
|  | Initiated insulin: n, % | C: n=3, 5%, Tx1: n=23, 23% |  |
| STeP,  Fisher (2011), Polonsky (2011)^a^, Polonsky (2011)^b^, Fisher (2012) [61-64] | Treatment modification recommended: medicine or lifestyle % | 1m, C: 28%, Tx1: 76%  3m, C: 35%, Tx1: 62%  6m, C: 22%, Tx1: 63%  4m, C: 19%, Tx1: 56%  12m, C: 11%, Tx1: 48% | Between groups:  p<.001  p<.001  p<.001  p<.001  p<.001 |
|  | Initiated insulin: n | 12m, C: 23, Tx1: 42 | Between groups: p=0.046 |
| ROSES,  Franciosi (2011) [66] | Diabetes medication/ dose change: n, % | C: n=9, 56%, Tx1: n=16, 35% | Between groups: NS |
| **Prospective observational single group study** | | | |
| ROSSO, Kempf (2010), Kempf (2012) [33,34] | OHA prescription: change n | Tx1: n=4 | Within group: NS |

C= Control, Tx1= Treatment 1, Tx2= Treatment 2, Tx3= Treatment 3, NR= not reported, NS= not significant, OHA= Oral Hypoglycaemic Agent, * per protocol

## Supplementary Table S4: Behavioral and psychosocial outcomes at baseline and follow up, change and difference at follow-up

| **Study Name (if specified)**  **Author name/s, year** | **Instrument used: unit reported** | **Baseline score** | **End of trial score** | **Change from baseline (within group)** | **Difference between groups at follow up (95% CI)** |
| --- | --- | --- | --- | --- | --- |
| **General health status** | | | | |  |
| ROSSO  Kempf (2010), Kempf (2012) (33,34)* | SF-36^  Physical health (PH)  Mental health (MH) | 12 weeks  Tx1 PH= 63.4 (62.0, 64.3)  Tx1 MH= 65.6 (64.6, 66.9) | 12 weeks  Tx1: PH= 67.9 (66.7, 68.9)  Tx1: MH= 70.2 (69.1, 71.3) | 12 weeks  Significant increase in PH and MH (p<0.001)  2 years  Improvements in PH not maintained, but improvements in MH maintained (p<0.05) | NA |
| ZODIAC  Kleefstra (65) | SF-36  Physical health (PH)  Mental health (MH) | PH  C: 48.5±10.6  Tx1: 42.2±10.4  MH  C: 50.6±10.6  Tx1: 55.5±7.4 | PH  C: 47.9±7.9  Tx1: 44.3±9.8  MH  C: 51.6±7.7  Tx1: 53.1±9.5 | NR | C vs Tx1 (PH) = -0.0 (-5.2, 5.1)  C vs Tx1 (MH)= -1.4 (-6.6, 3.7)  NS |
| DiGEM  Farmer (2007), French (2008), Simon (2008) (20,40,51)* | EQ-5D (Utility values) | C: 0.799±0.023  Tx1: 0.781±0.022  Tx2: 0.807±0.024 | C: 0.798±0.034  Tx1: 0.755±0.024  Tx2: 0.733±0.024 | C: -0.001 (95% CI, -0.060 to 0.059)  Tx1: -0.027 (95% CI, -0.069 to 0.015)  Tx2: -0.075 (95% CI, -0.119 to -0.031), p<0.05  Significant | C vs Tx1: -0.029 (95% CI, 0.084 to 0.025), p>.05  C vs Tx2: -0.072 (95% CI, -0.127 to -0.017), p<0.05  Significant |
| **General emotional well-being** | | | | | |
| DiGEM  Farmer (2007), French (2008), Simon (2008) (20,40,51)* | W-BQ12 | C: 25.1±6.3  Tx1: 25.2±6.3  Tx2: 24.3±6.8 | C: 25.9±5.8  Tx1: 24.9±6.4  Tx2: 24.5±7.0 | NR | NS, p=0.38 |
| Li (2016) (35^) | WHO-5 | NR | NR | Tx1: 40  Tx2: 65  Tx3: 60 | Tx1 vs Tx2/T3 = p<.05 |
| SMBGb*  Schwedes (2002),  Siebolds (2006) (36,37) | W-BQ22^ | C: 50.66±9.46  Tx1: 50.52±8.74 | C: 52.55±10.47  Tx1: 54.03±8.24 | C: 1.75±7.33  Tx1: 3.58±7.01 | NS, p=0.053 |
| STeP  Fisher (2011), Polonsky (2011)^a^, Polonsky (2011)^b^, Fisher (2012)(61-64) | WHO-5 | C: 58.0±20.7  Tx1: 57.3±23.6 | C: 62.0±20.8  Tx1: 62.0±20.8 | C: p<0.007  Tx1: p<0.0001 | ITT: NS  PP: p<0.04 |
| ZODIAC  Kleefstra (2010) (65) | WHO-5 | C: 71.0±17.9  Tx1: 68.0±20.7 | C: 76.3±11.4  Tx1: 74.4±14.5 | NR | CTx1 = -0.6 (-8.2, 7.0)  NS |
| **Depressive symptoms** | | | | |  |
| Cox (2020) (49) | PHQ-9 | NR | NR: | C: -0.55±4.11  Tx1: 0.00±4.42  Tx2: -1.74±5.13 | NS  p=0.298 |
| Li (2016) (35)* | CES-D^ | Nr | Nr | Tx1: -6.5  Tx2: -7.5  Tx3: -11  NS | NS |
| ROSSO  Kempf (2010), Kempf (2012) (33,34)* | CES-D(Mean±SE):  % likely depressed^ | Tx1: 14.9 (14.5, 15.2)  Tx1: 7.9%^#^ | 12 weeks  Tx1: 14.2 (13.9, 14.4)  12 weeks  Tx1: 4.8%  2 years  Tx1: 7.0% | No change in % likely depressed at 12 weeks or 2-year follow-up |  |
| IN CONTROL  Malanda (2016) (54) | PHQ-9 | C: 3.6±5.1  Tx1: 2.6±3.4  Tx2: 4±4.4 | C: 3.1±4.7  Tx1: 3.1±3.7  Tx2: 4.1±4.6 | C: -0.5±3.2  Tx1: -0.4 ±2.8  Tx2: 0.5±2.0  NS | C vs Tx1 = -0.2 (CI= -0.7 to 0.4)  C vs Tx2: -0.8 (CI= 1.8 to 0.3)  Tx1 vs Tx2: 0.6 (CI=-0.4 to1.7)  NS |
| STeP  Fisher (2011), Polonsky (2011)^a^, Polonsky (2011)^b^, Fisher (2012) (61-64) | PHQ-8 | C: 5.85±5.4  Tx1: 6.54±6.0 | C: 5.05±0.35  Tx1: 4.54±0.33 | C: -1.14  Tx1: -1.66  P<0.001 | C vs Tx1: 0.28  NS |
| **Diabetes distress** | | | | |  |
| Cox (2020) (49) | DDS (Emotional subscale)  DDS (Regimen subscale) | NR  NR | NR  NR | C: -0.27±1.13  Tx1: -0.03±0.70  Tx2: -0.44±0.96  C: -0.74±1.42  Tx1: -0.43±1.14  Tx2: -0.67±1.17 | NS  p=0.344  NS  p=0.817 |
| IN CONTROL  Malanda (2016) (54) | PAID | C: 9.1±11.0  Tx1: 14.2±14.7  Tx2: 6.7±6.5 | C: 7.9±9.0  Tx1: 14.2±15.1  Tx2: 7.7±10.1 | C: -1.2±10.8  Tx1: 0.0±14.8  Tx2: 1.0±8.0  NS | C vs Tx1 = -2.0 (CI= -4.1 to 0.1)  C vs Tx2: -0.9 (CI= -4.4 to 2.5)…  Tx1 vs Tx2: -2.2 (CI= -7.1 to 2.7)  NS |
| Li (2016) (35)^ | PAID^ | NR | NR | Tx1: -1.8  Tx2: -3.2  Tx3: -2.8 | Tx1 vs Tx2/T3 = p<.05 |
| STeP  Fisher (2011), Polonsky (2011)a, Polonsky (2011)b, Fisher (2012) (61-64) | DDS | C: 2.25±0.06  Tx1: 2.41±0.06 | C: 1.93±0.07  Tx1: 1.78±0.06 | C: -0.40 (p<0.001)  Tx1: -0.55 (p<0.001) | C vs Tx1: 0.12  NS |
| **Diabetes-specific quality of life** | | | | |  |
| PRISMA  Bosi (2013), Russo (2016) (58,59) | DsQOL: Impact, Satisfaction, Worry | Impact  Tx1: 32.1±7.30  Tx2: 32.4±7.09  Satisfaction  Tx1: 34.3±7.63  Tx2: 33.0±7.38  Worry  Tx1: 9.1±3.27  Tx2: 8.6±2.73 | NR | Impact  Tx1: -0.65±0.49  Tx2: -0.66±0.49  Satisfaction  Tx1: -1.75±0.53  Tx2: -2.31±0.54  Worry  Tx1: -0.47±0.21  Tx2: -0.47±0.22 | Impact  NS (-0.006±0.38, p= 0.9864)  Satisfaction  NS (-0.56±0.44, p= 0.2099)  Worry  NS (0.004±0.16, p= 0.9784) |
| Kan (2017) (55) | Cn-ADDQoL | NR | NR | NR | General QoL single overview item: Significant Insulin-treated: p=0.021 Non-insulin-treated: p<0.001 Diabetes-specific QoL single overview items: NS |
| **Diabetes-specific treatment satisfaction** | | | | |  |
| DiGEM  Farmer (2007), French (2008), Simon (2008) (20,40,51)* | DTSQ | C: 29.3±6.8  Tx1: 29.7±5.4  Tx2: 29.4±6.5 | C: 30.0±5.3  Tx1: 30.1±5.5  Tx2: 29.7±5.6 | NR | NS (p=0.93) |
| IN CONTROL  Malanda (2016) (54) | DTSQ | C: 29.8±4.6  Tx1: 27.6±6.3  Tx2: 31.0±4.1 | C: 30±4.0  Tx1:28.8±5.4  Tx2: 29.0±6.7 | C: 0.2±4.2  Tx1: 1.2±6.7  Tx2: -2.03±6.2  NS | C vs Tx1 = 0.2 (CI= -0.7 to 1.1)  C vs Tx2: 1.7 (CI= -0.3 to 3.7)  Tx1 vs Tx2: -1.2 (CI= -3.6 to 1.3)  NS |
| SMBG b*  Schwedes (2002), Siebolds (2006) (36,37) | DTSQ^ | C: 26.95±6.6  Tx1: 27.58±7.1 | C: 30.57±5.54  Tx1: 31.1±4.78 | C: 3.6±7.63  Tx1: 3.52±7.19 | NS, p= 0.9 |
| ZODIAC  Kleefstra (2010) (65) | DTSQ | C: 30.7±4.2  Tx1: 29.3±4.8 | C: 30.7±4.0  Tx1: 32.1±3.8 | NR | C vs Tx1 = 1.2 (-1.6, 4.1)  NS |
| **Diabetes-specific self-efficacy** | | | | |  |
| Cox (2020) (49) | DES | NR | NR | C: 2.70±4.35  Tx1: 3.30±6.17  Tx2:3.24±4.89 | NS  p=0.824 |
| IN CONTROL  Malanda (2016) (54) | CIDS-2 | C: 78.1±15.1  Tx1: 80.9±10.2  Tx2: 84.4±9.8 | C: 78.0±12.2  Tx1: 79.3±12.2  Tx2: 79.0±15.4 | C: -0.14±13.1  Tx1: -1.6±10.3  Tx2: -5.4±11.9  NS | C vs Tx1 = 0.6 (CI= -2.0 to 2.1)  C vs Tx2: 2.8 (CI= -2.3 to 7.9)  Tx1 vs Tx2: -3.3 (CI= -7.9 to 1.3)  NS |
| Greenwood (2015) (53) | DES-SF | C: 3.5 (3.3, 3.8)  Tx1: 3.8 (3.2, 4.4) | NR | NR | NR |
| STeP (Protocol adherent)  Fisher (2011), Polonsky (2011)^a^, Polonsky (2011)^b^, Fisher (2012) (61-64) | CIDS-2 (mean±SE) | C: 79.6±0.75  Tx1: 79.6±0.75 | C: 82.1±1.1  Tx1: 85.3±1.1 | C: (p<0.001)  Tx1: (p<0.001) | ITT: NS  PP: p<0.05 |
| Li (2016) (35)^^^ | PDSMS^ | NR | NR | Tx1: -1.3  Tx2: -1.8  Tx3: -1.8 | NS |
| **Diabetes self-care behaviors (self-reported)** | | | | |  |
| DiGEM  Farmer (2007), French (2008), Simon (2008) (20,40,51)* | SDSCA: general diet and exercise subscales  Medication-taking: MARS | General diet C: 5.2±1.8  Tx1: 5.0±1.8  Tx2: 5.2±1.8  Exercise  C: 3.3±2.1  Tx1: 3.2±2.2  Tx2: 3.4±2.2  Medication taking  C: 24.0±1.6  Tx1: 23.9±1.6  Tx2: 24.0±1.3 | General diet C: 5.6±1.5  Tx1: 5.5±1.3  Tx2: 5.1±1.9  Exercise  C: 4.0±2.2  Tx1: 3.6±2.2  Tx2: 3.9±2.4  Medication taking  C: 24.1±2.0  Tx1: 24.0±1.4  Tx2: 24.0±1.3 | NR | General diet: Significant  C vs Tx1 vs Tx2  (p=0.014)  C vs Tx1: 0.12 (–0.33 to 0.57; d=0.06)  C vs Tx2: –0.50 (–1.00 to 0.01; d=0.23  Exercise  NS (p=0.45)  Medication taking  NS (p=0.91) |
| Greenwood (2015) (53) | SDSCA: general diet, exercise and medication taking subscales  Mean (95% CI) | General Diet  C: 3.7 (3.2, 4.3)  Tx1: 3.7 (2.4, 5.0)  Exercise  C: 2.4 (1.7, 3.1)  Tx1: 2.7 (1.1, 4.3)  Medication taking  C: 6.5 (6.0, 7.0)  Tx1: 6.2 (4.9, 7.0) | NR | NR | NR |
| Nishimura (2017) (57) | SDSCA: diet, exercise and medication subscales | NR | NR | General diet  C: 0.83 (95% CI: 0.44 to 1.23), p<0.001  Tx1: -0.03 (95% CI: -0.44 to 0.39)  Exercise  C: 0.72 (95% CI: 0.11 to 1.33), p<0.05  Tx1: 0.64 points (95% CI: −0.15 to 1.44)  Medication  NR | General diet  Significant  0.86 (95% CI: 0.30 to 1.42), p<0.01  Exercise  NS  Medication  NS |
| STeP (Protocol adherent)  Fisher (2011), Polonsky (2011)^a^, Polonsky (2011)^b^, Fisher (2012) (61-64) | IPAQ short form | NR | NR | NR | NR |
| **Other psychological process and outcomes** | | | | |  |
| DiGEM  Farmer (2007), French (2008), Simon (2008) (20,40,51)* | Beliefs about medication: BMQ  Illness perceptions: IPQ-R | BMQ: Necessity  C: 19.1±2.3  Tx1: 18.5±3.2  Tx2: 18.719.4±2.82.9  BMQ: Concerns  C: 13.7±2.9  Tx1: 13.6±3.9  Tx2: 13.3±3.8  IPQ-R: Identity  C: 2.2±2.8  Tx1: 1.7±2.2  Tx2: 1.8±2.1  IPQ-R: Timeline – acute/ chronic  C: 24.5±4.1  Tx1: 24.4±4.0  Tx2: 25.3±3.5  IPQ-R: Timeline – cyclical  C: 10.4±3.3  Tx1: 10.4±3.0  Tx2: 10.6 ±2.9  IPQ-R: Consequences  C: 17.3±3.9  Tx1: 16.9±3.7  Tx2: 17.2±3.4  IPQ-R: Personal control  C: 24.1±3.1  Tx1: 24.2±2.7  Tx2: 24.7±3.1  IPQ-R: Treatment control  C: 18.2±1.9  Tx1: 18.3±2.0  Tx2: 18.6±2.0  IPQ-R: Illness coherence  C: 16.9±4.1  Tx1: 16.6±4.5  Tx2: 17.1±4.1  IPQ-R: Emotional representations  C: 15.3±4.3  Tx1: 15.1±4.7  Tx2: 15.6±4.7 | BMQ: Necessity  C: 18.9±2.4  Tx1: 18.6±2.9  Tx2: 19.4±2.8  BMQ: Concerns  C: 13.3±3.2  Tx1: 13.1±3.5  Tx2: 13.4±3.9  IPQ-R: Identity  C: 1.7±2.2  Tx1: 2.0±2.4  Tx2: 1.8±2.0  IPQ-R: Timeline – acute/ chronic  C: 25.0±3.6  Tx1: 24.6±4.3  Tx2: 25.4±4.0  IPQ-R: Timeline – cyclical  C: 10.0±3.1  Tx1: 10.4±3.1  Tx2: 10.7±3.1  IPQ-R: Consequences  C: 16.8±4.3  Tx1: 17.4±4.2  Tx2: 18.3±3.8  IPQ-R: Personal control  C: 24.3±2.8  Tx1: 24.1±3.5  Tx2: 25.0±3.6  IPQ-R: Treatment control  C: 17.8±2.0  Tx1: 18.3±1.9  Tx2: 18.1±2.5  IPQ-R: Illness coherence  C: 17.4±4.4  Tx1: 17.8±4.1  Tx2: 18.2±4.1  IPQ-R: Emotional representations  C: 15.0±4.0  Tx1: 15.3±4.6  Tx2: 15.4±4.8 | NR | BMQ: Necessity  NS (p=0.28)  BMQ: Concerns  NS (p=0.75)  IPQ-R: Identity  NS (p=0.054)  IPQ-R: Timeline – acute/ chronic  NS (p=0.61)  IPQ-R: Timeline – cyclical  NS (p=0.33)  IPQ-R: Consequences  Significant (p=0.004)  IPQ-R: Personal control  NS (p=0.30)  IPQ-R: Treatment control  NS (p=0.29)  IPQ-R: Illness coherence  NS (p=0.36)  IPQ-R: Emotional representations  NS (p=0.58) |
| Greenwood (2015) (53) | Diabetes Knowledge Test: DKT | Diabetes Knowledge Test: DKT  Mean (95% CI) | C: 12.0 (11.3, 12.6)  Tx1: 12.4 (10.9, 13.9) | NR | NR |
| Cox (2020) (49) | Glucose Monitoring Satisfaction Scale (GMSS) | NR | NR | C: 0.32±0.52  Tx1: 0.33±0.57  Tx2:0.46±0.57 | NS  p=0.27 |
| PRISMA  Bosi (2013), Russo (2016) (58,59) | Locus of Control: LOC | Chance  Tx1: 16.3±6.81  Tx2: 16.1±6.09  Internal  Tx1: 30.6±4.48  Tx2: 30.6±4.56  Others  Tx1: 25.6±5.01  Tx2: 25.7±5.36 | NR | Chance  Tx1: -0.034±0.54  Tx2: -0.99±0.57  Internal  Tx1: 0.03±0.39  Tx2: 0.09±0.39  Others  Tx1: -0.18±0.41  Tx2: -0.41±0.43 | Chance  Significant (-0.96±0.44, p= 0.0309)  Internal  NS (0.06±0.31, p=0.8394)  Others  NS (-0.22 (0.34), p=0.5128) |
| STeP  Fisher (2011), Polonsky (2011)^a^, Polonsky (2011^)^b, Fisher (2012) (61-64) | Autonomous motivation: DRAM | C: 6.11±0.05  Tx1: 6.11±0.05 | C: 6.18±0.07  Tx1: 6.39±0.07 | ITT NS, PP both groups had significant increases in scores | ITT: NS  PP: Significant, p<0.05 |
| ZODIAC  Kleefstra (2010) (65) | Diabetes Symptoms (DSC-R), Median (IQR) | C: 0.7 (0.4, 1.0)  Tx1: 0.5 (0.2, 1.0) | C: 0.9 (0.3, 1.4)  Tx1: 0.4 (0.3, 1.1) | NR | C vs Tx1: -0.1 (-0.5, 0.3)  NS |

Data are mean±SD unless otherwise reported. ^ Scores determined using web plot digitiser, ^#^ % cases meeting criteria for a diagnosis of depression, *Per protocol data reported. C= Control, Tx1= Treatment 1, Tx2 = Treatment 2, Tx3= Treatment 3, NR=not reported, NS=not significant, ITT= intention to treat, PP= per protocol
BMQ: Beliefs about Medicines Questionnaire, CES-D: Center for Epidemiologic Studies Depression scale, CIDS-2: Confidence in Diabetes Self-Care questionnaire, CN-ADDQoL: Chinese Normal Audit of Diabetes-Dependent Quality of Life, DDS: Diabetes Distress Scale, DES-SF: Diabetes Empowerment Scale short form, DKT: Diabetes Knowledge Test, DRAM: Diabetes-Related Autonomous Motivation assessed by 6 item subscale of Treatment Self-Regulation Scale, DSC-r: Diabetes Symptoms Checklist revised, DsLOC: Diabetes-specific Locus of Control, DSQoL: Diabetes-specific Quality of Life, DTSQ: Diabetes Treatment Satisfaction Questionnaire, EQ-5D: EuroQol EQ-5D, IPAQ: International Physical Activity Questionnaire, IPQ-R: Illness Perceptions Questionnaire revised, MARS: Medication Adherence Report Scale, PAID: Problem Areas In Diabetes scale, PDSMS: Perceived Diabetes Self-Management Scale, PHQ-8/9: Patient Health Questionnaire- 8/9 item, PWQ: Patient Well-being Questionnaire, SDSCA: Summary of Diabetes Self-Care Activities, SF-36: Short-Form 36 Health Survey questionnaire, W-BQ12: Well-Being Questionnaire 12-item, WHO-5: WHO five-item Well-being Index

## Supplementary Table S5: Comparison of RCTs included in current and prior published reviews

| **Studies included in this review**  (sSMBG v no SMBG^i^ / uSMBG^ii^ / CGM^iii^, pub. ≤ 2020) | **Malanda et al 2012** (17)  (SMBG v no SMBG/usual care,  pub. ≤2011) | **Zhu et al 2016** (31)  (SMBG v no SMBG/usual care, pub. ≤2015) | **Mannucci et al. 2018** (29)  (SMBG v no SMBG & sSMBG v uSMBG, pub. ≤ 2015) | **Chircop et al 2021** (30) (SMBG v usual care & sSMBG v uSMBG, pub. ≤2020) | **Effective** (HbA1c) |
| --- | --- | --- | --- | --- | --- |
| SMBG^b^  Schwedes (2002), Siebolds (2006) (36,37)* | X^i^ | X^i^ | X^i^ | X ^i^ | Yes |
| Davidson (2005) (50)* | X^i^ | X^i^ | X^i^ | X ^i^ | No |
| DiGEM  Farmer (2007), French (2008), Simon (2008) (20,40,51)* | X^i^ | X^i^ | X^i^ | X ^i^ | No |
| DINAMIC 1  Barnett (2008) (52)* | X^i^ | X^i^ | X^i^ | X ^i^ | Yes |
| St Carlos  Duran (2010) GarcíaDeLaTorre (2013) (46,60)* | X^ii^ | X^ii^ | X^ii^ | X ^ii^ | Yes |
| ZODIAC  Kleefstra (2010) (65)* | X^ii^ | X^ii^ | X^ii^ | X ^ii^ | No |
| Bonomo (2010) (48) |  |  |  |  | No |
| ROSES  Franciosi (2011) (66)* | X^i^ | X^i^ | X^i^ | X ^i^ | Yes |
| STeP  Fisher (2011), Polonsky (2011)^a^, Polonsky (2011)^b^, Fisher (2012) (61-64)* |  |  | X^ii^ | X ^ii^ | Yes |
| ROSSO international  Kempf (2013) (45)* |  | X^i^ |  |  | No |
| PRISMA  Bosi (2013), Russo (2016) (58,59) |  |  | X^ii^ | X ^ii^ | No |
| Greenwood (2015) (53)* |  |  |  |  | No |
| Ngaosuwan (2015) (56) |  |  |  |  | Yes |
| IN CONTROL  Malanda (2016) (54)* |  | X ^ii^ |  | X ^ii^ | No |
| Li (2016) (35) |  |  |  |  | Yes |
| Nishimura (2017) (57)* |  |  |  | X ^ii^ | No |
| Kan (2017) (55) |  |  |  | X ^ii^ | No |
| SMBG^a^  Parsons (2019) (41)* |  |  |  | X ^i^ | Yes |
| Cox (2020) (49)* |  |  |  |  | No |
| Bergenstal (2022) (47) |  |  |  |  | No |

SMUG: Self-monitored urine glucose, ^I^ SMBG v no SMBG; ^ii^ sSMBG v. uSMBG; ^iii^ sSMBG v. CGM *included in meta-analysis

# Supplementary Figures


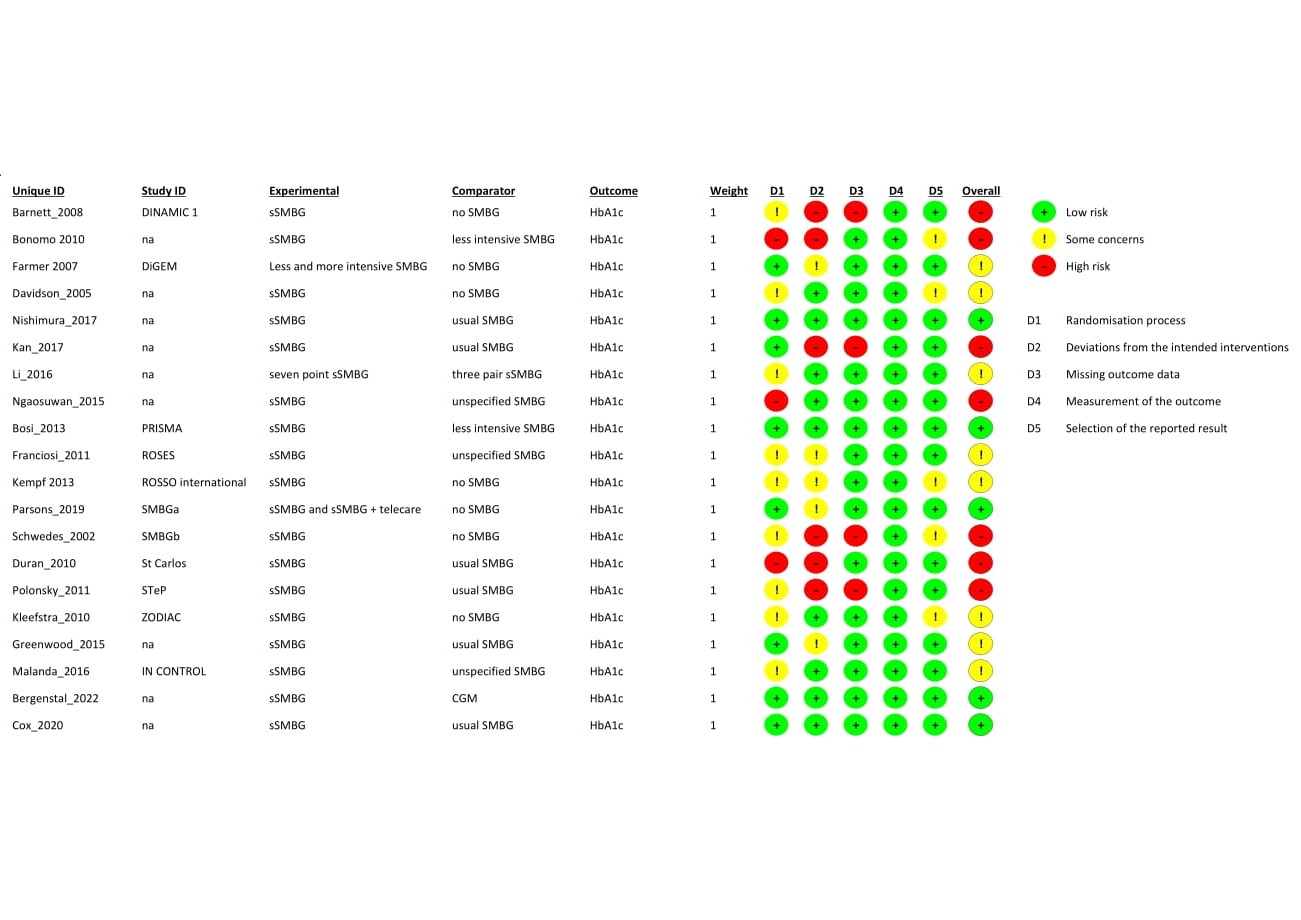


## Supplementary Figure S1: Risk of bias assessments for RCTs (HbA1c)


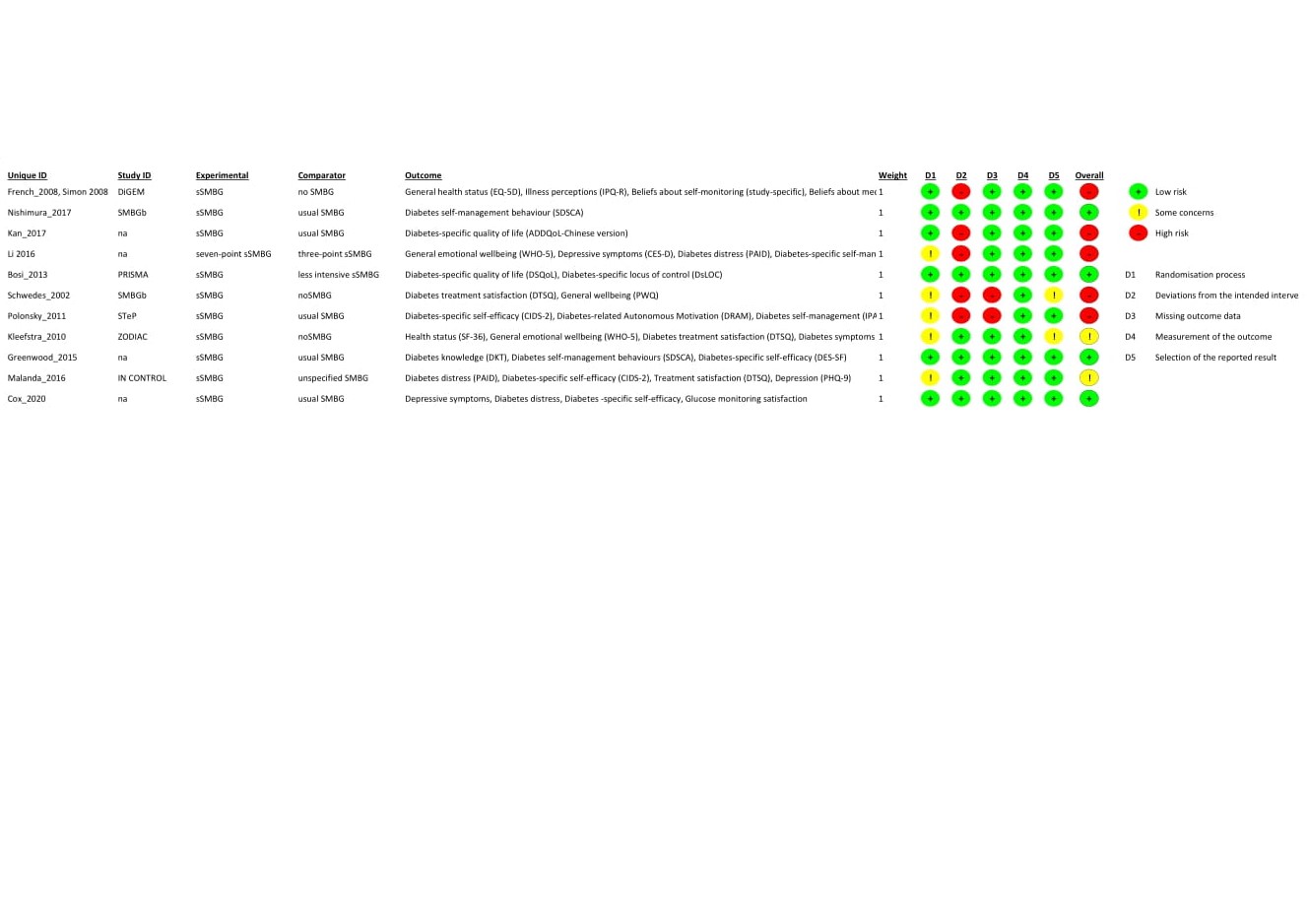


## Supplementary figure S2: Risk of bias assessments for RCTs (Psychosocial outcomes).
